# Small-scale mobility fostering the interaction networks of Patagonian (Argentina) hunter-gatherers during the Late Holocene: Perspectives from strontium isotopes and exotic items

**DOI:** 10.1371/journal.pone.0281089

**Published:** 2023-02-15

**Authors:** Alejandro Serna, Clément P. Bataille, Luciano Prates, Emiliano Mange, Petrus le Roux, Domingo C. Salazar-García

**Affiliations:** 1 BioArCh, Department of Archaeology, University of York, York, North Yorkshire, United Kingdom; 2 División Arqueología, Facultad de Ciencias Naturales y Museo, Universidad Nacional de La Plata, La Plata, Buenos Aires, Argentina; 3 Department of Earth and Environmental Sciences, University of Ottawa, Ottawa, Ontario, Canada; 4 Consejo Nacional de Investigaciones Científicas y Técnicas, Buenos Aires, Argentina; 5 Department of Geological Sciences, University of Cape Town, Cape Town, Western Cape, South Africa; 6 Departament de Prehistòria, Arqueologia I Història Antiga, Universitat de València, València, València, Spain; Northeast Normal University, CHINA

## Abstract

During the Late Holocene, hunter-gatherer interaction networks significantly grew in intensity and extension across Patagonia. Although this growth is evidenced by the increased flow of exotic items across the region, the mechanisms behind these strengthening social networks remain unclear. Since evidence suggests that some individuals might have performed long-distance trips, this article aims to address the potential relationship between these individuals and the flows of exotic items in North Patagonia. We analyzed 54 enamel teeth for strontium isotopes and reconstructed their probable mobility using mixed-effect models and isotope-based geographic assignments. We inferred population and individual mobility trends and compared them against the flow of exotic items built from a standardized compilation. Our results indicate that most individuals have isotopic composition compatible with residence within their burial and surrounding areas. However, a few individuals show isotopic composition incompatible with their burial areas, which suggests axes -from the burial location to the most likely isotope integration area- of extraordinary mobility. At the same time, the flows of exotic items overlap with these axes around the eastern sector of the study area suggesting that this location could have been a central point of convergence for people and items. We argue that small-scale socially driven mobility could have played a relevant role as a general mechanism of interaction that fostered and materialized Patagonian interaction networks during the Late Holocene.

## Introduction

**Fig 1:**
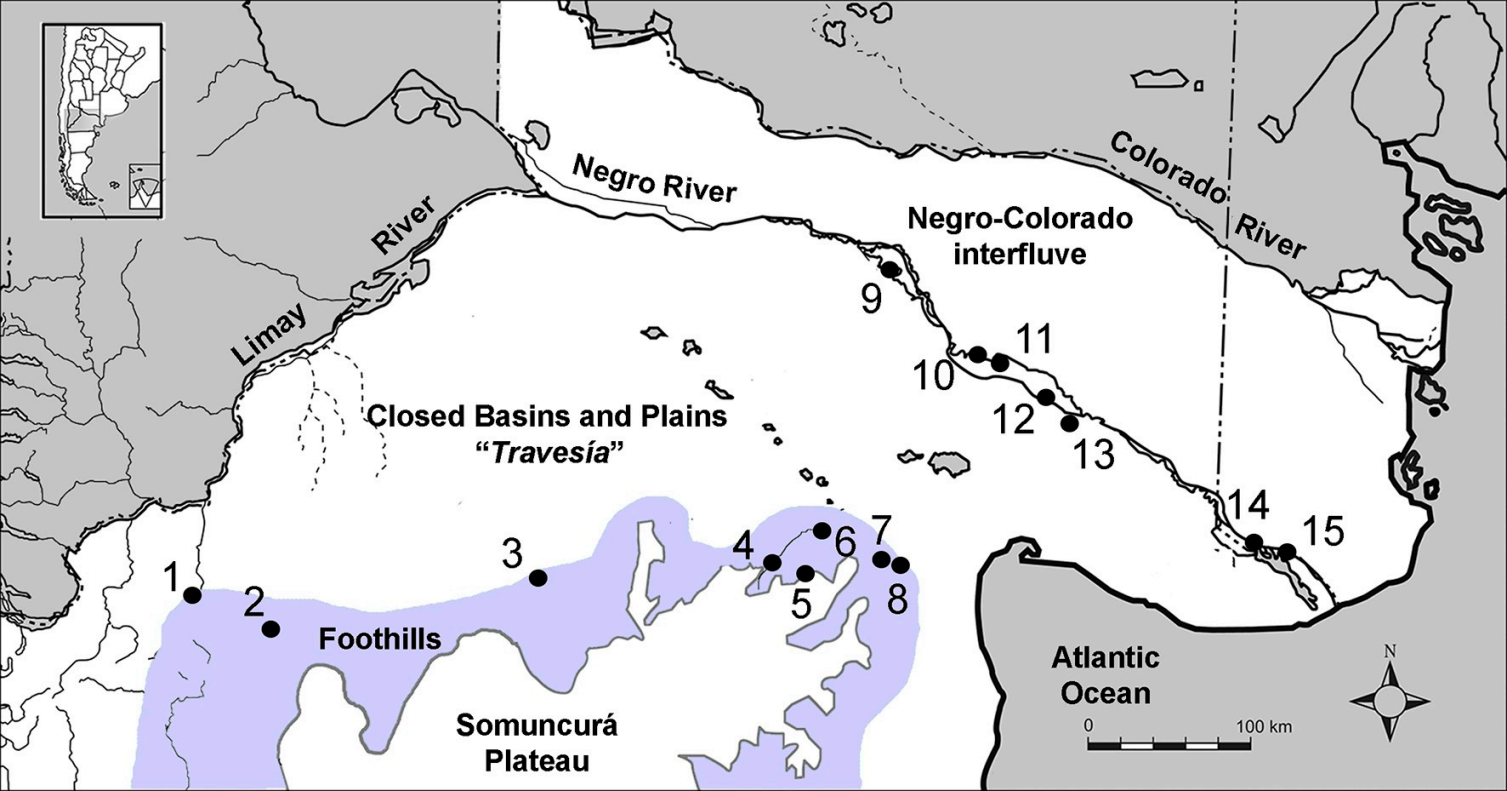
Study area and locations of the archaeological sites with human remains analyzed in this study (lat. -42° to -38° S, long. -70° to -62° W). From Somuncurá Foothills (SF): 1-Comallo; 2-Matadero Jacobacci; 3-Colitoro; 4-Chipauquil; 5-Paja Alta; 6-Valcheta; 7-Aguada Cecilio; 8-Cueva Galpón. From Negro River (NR): 9- Museo Beltrán, Pomona; 10-Negro Muerto 2 and 3; 11-La Victoria 5; 12-Caitacó, 13-Loma de los Muertos; 14-San Javier; 15-Laguna del Juncal. Adapted from A. Serna Unpublished PhD thesis under a CC BY license, with permission of the author, original copyright 2018.

Human mobility has been a relevant issue in archaeological research for decades and most of the attention has been placed on discussing large-scale and/or organized population mobility, which are usually associated with clear material expressions (e.g., seasonal rounds: [1, 2]; invasions: [3–5]; migrations and diasporas: [6–9]). Less attention has been paid to small-scale mobility (i.e., one or few individuals), that might be regular but discrete in terms of archaeological evidence [10]. In hunter-gatherers, this mobility has been mainly related to daily subsistence tasks [11–13], but also to social aims such as visit acquaintances, relieving boredom and/or exchange [14–16]. “Exotic” items might represent a key archaeological expression of this small-scale socially driven mobility since they are powerful vehicles to model and materialize networks [17–19]. By combining an item-based approach with isotope analysis on human remains [20, 21], we will focus on detecting this mobility in North Patagonia (Fig 1).

North Patagonia is a region of the Southern Cone of South America where hunter-gatherer interaction networks grew more extensive and intense during the Late Holocene (ca. 3000–250 years BP) (e.g., [22–25]). A context of fluid interactions has been proposed from human mobility [26], flow of stylistic expressions (portable and rock art: [27, 28]; pottery: [29]; egg shells: [30]; cranial modification: [31]), and largely by exotic item circulation (lithic, mollusks and ornaments: [23–25, 32–34]; pottery: [35, 36]; ritual artefacts: [22]; edibles: [37, 38]). Previous research based on oxygen isotopes (δ^18^O) on human enamel and water sources in this area showed that the drinking water values of some individuals did not match with the water baseline built from the main available sources [26]. The presence of these individuals with unidentified drinking water sources might be related to “extraordinary” mobility patterns, which in this context would be characterized by long-distances and/or potential connections with distinct environments relative to their burial place. Several questions derive from this hypothesis: is there a causal relationship between these extraordinary mobility patterns and the flow of items? Could small-scale mobility be operating as a general mechanism of interaction? Who moved and carried these items across the landscape the most, large groups or a few individuals?

Here we tackle these questions by studying human paleomobility and exotic item flows in northern Patagonia, Argentina (Fig 1). First, we explore trends of mobility -with particular attention to individuals with unidentified drinking water sources- by analyzing strontium isotopes in human enamel and performing probabilistic geographic assignments. Then, we evaluate the potential role of small-scale mobility by comparing trends of mobility with the flow of exotic items reconstructed from a standardized compilation. If small-scale mobility worked as a relevant mechanism of interaction in these societies, we would expect that the individuals with extraordinary mobility show compatibility with regions where the exotic items come from or, similarly, areas where item flow most likely occurred (i.e., an “overlap” between human mobility and item flow).

## Materials and methods

### Strontium and sample background

The strontium isotope ratio (^87^Sr/^86^Sr) is one of the most common isotope tracers used to address past mobility. This is because bioavailable Sr is propagated predictably from the geosphere to the biosphere. The ^87^Sr/^86^Sr of mineralized tissues is directly related to the ^87^Sr/^86^Sr of bioavailable Sr on the dietary catchment exploited during the time of tissue development (see reviews in [39, 40]). Hence, the comparison between the isotope ratios of a given sample against a baseline is necessary and can be achieved by different quantitative approaches [41]. Given that the study region counts with a high-resolution baseline [42], this work will adopt a continuous probabilistic approach to perform the geolocation on the archaeological samples.

The samples analyzed in this work come from northern Patagonia, Argentina (Fig 1). The arid/semi-arid conditions that reign in most of this region are the result of the Andes intercepting the humid air masses from the Pacific, leaving them with less moisture as they pass over the mountains to the east [43, 44]. Regardless of some local variations, paleoclimate reconstructions show that these general conditions have prevailed for the last ca. 3000 years BP [45]. The scarcity and distribution of freshwater sources have defined a fragmented environment characterized by large “dry lands” with very low availability of temporary water bodies intersected by highly productive “wet stripes” defined by rivers and streams (Fig 1) [46, 47]. These drylands are the Negro-Colorado interfluve and the area located south of the Negro River valley (“*travesía”*), which is known as one of the harshest landscapes in Patagonia [48]. These dry lands are delineated by the Colorado and Negro river valleys, two of the most relevant permanent watercourses in Patagonia, and by the streams draining from the Somuncurá Foothills [46, 47].

Human burials occurred in natural niches and rock mound burials (i.e., *chenques*), and -more usual- they are located in elevated dunes near paleochannels [25, 49–52]. The spatial association of human burials with remains of residential occupations (e.g., faunal remains, lithic flakes, and pottery sherds) is one of the most common archaeological patterns in the region, and it has been understood as the result of the recurrent use of the same location through time [49]. This selection would have been based on positive features of the landscape and the knowledge about these locations probably transmitted as a part of the interaction networks that took place during the Late Holocene among North Patagonian and neighbouring people [49]. Apart from the circulation of items and stylistic expressions, cranial modifications could have also played a role as another correlate of these networks that implied material and non-material exchange at a macro-regional scale (see [31]).

The analyzed samples come from hunter-gatherer sites located at the Negro River and the Somuncurá Foothills (Fig 1). As it has been recognized in neighbouring regions [53], they buried their dead in a non-standardized way, including primary, secondary, simple and multiple modes, being funerary goods rarely involved [49, 51]. Some of the analyzed individuals were dated in the Late Holocene (Table 1), which is congruent with the majority of the archaeological sites in the study area [23, 25, 46, 51]. These remains are housed at the División Arqueología, Museo de La Plata, Universidad Nacional de La Plata (Argentina).

**Table 1:** Isotope data of the analyzed samples.

Loc.	Site	Ind.	^14^C (years BP)	Sex	Tooth^a^	Mineral.^b^	Lab code (UCT)	^87^Sr/^86^Sr	±2σ	Δ^87^Sr/^86^Sr	[Sr] (ppm)	1/[Sr]	Δ1/[Sr]	Reference (^14^C)
NR	13	LM_1	2088 ± 46	M	LR-M3	late*	18789	0.707238	0.000016	-	256	0.0039	-	[55]
		LM_3	2718 ± 47	F	UL-M3	late*	18791	0.707267	0.000010	0.0005	222	0.0045	0.0008	[55]
				F	UL-M2	early*	18792	0.707785	0.000013	-	273	0.0037	-	-
		LM_4		Indet.	UL-M2	early	18793	0.706197	0.000010	-	169	0.0059	-	-
	10	NM2_1		F	UL-M3	late	18794	0.706151	0.000010	0.0000	332	0.0030	0.0006	-
				F	LR-M2	early	18795	0.706189	0.000013	-	413	0.0024	-	-
		NM2_2	1586 ± 47	F	UR-M3	late	18796	0.706372	0.000013	0.0002	218	0.0046	0.001	[56]
				F	LL-M2	early	18797	0.706239	0.000014	-	277	0.0036	-	-
		NM2_3	1637 ± 48	F	LR-M2	early*	18798	0.706054	0.000010	-	234	0.0043	-	[56]
		NM3_1		M	UR-M3	late	18799	0.706154	0.000011	0.0000	257	0.0039	0.0001	-
				M	UL-M2	early	18800	0.706169	0.000014	-	264	0.0038	-	-
		NM3_2		M	LR-M3	late*	18801	0.706120	0.000011	0.0001	233	0.0043	0.0011	-
				M	LL-M2	early	18802	0.706159	0.000010	-	185	0.0054	-	-
		NM3_3	1091 ± 35	M	UR-M3	late*	18803	0.706508	0.000011	0.0000	223	0.0045	0.0004	[38]
				M	UR-M2	early*	18804	0.706495	0.000014	-	204	0.0049	-	-
		NM3_4		M	LL-M3	late	18805	0.706124	0.000010	0.0001	182	0.0055	0.0004	-
				M	LR-M2	early	18806	0.706247	0.000011	-	197	0.0051	-	-
		NM3_5		F	LL-M3	late	18807	0.706154	0.000010	0.0000	200	0.0050	0.0018	-
				F	LL-M2	early*	18808	0.706180	0.000013	-	147	0.0068	-	-
	9	Po_1	986 ± 36	M	LR-M3	late	18809	0.706357	0.000012	0.0001	260	0.0038	0.0011	[25]
				M	LR-M2	early	18810	0.706300	0.000013	-	206	0.0049	-	-
	11	LV5_1	928 ± 39	F	UR-M3	late	18811	0.706756	0.000013	0.0001	334	0.0030	0.0003	[57]
				F	UR-M2	early	18812	0.706682	0.000011	-	305	0.0033	-	-
		LV5_2	868 ± 48	F	UL-PM2	early*	18813	0.706722	0.000013	-	229	0.0044	-	[57]
	9	MB_1		F	UL-M2	early	18817	0.706514	0.000011	-	221	0.0045	-	-
	12	Cai_1		M	UR-PM1	early	18818	0.706221	0.000013	-	164	0.0061	-	-
	14	SJ_C		F	UR-M2	early*	18819	0.707351	0.000012	-	187	0.0053	-	-
		SJ_J		M	UL-PM1	early*	18821	0.706927	0.000010	-	131	0.0076	-	-
		SJ_Ñ		M	UL-M2	early*	18824	0.708920	0.000012	-	206	0.0049	-	-
		SJ_GQ		Indet.	UL-M3	late*	18825	0.708907	0.000012	0.0018	155	0.0064	0.0004	-
				Indet.	UL-M2	early	18826	0.707131	0.000010	-	147	0.0068	-	-
	15	JunVal_1		Indet.	UL-M2	late*	18827	0.706850	0.000010	0.0000	154	0.0065	0.0003	-
				Indet.	UL-PM2	early*	18828	0.706890	0.000011	-	147	0.0068	-	-
		JunJa_1		F	UR-M3	late	18829	0.707024	0.000014	0.0001	176	0.0057	0.0002	-
				F	UR-M2	early*	18830	0.706947	0.000012	-	170	0.0059	-	-
		JunJa_2		M	UR-M3	late	18831	0.706920	0.000011	0.0003	260	0.0038	0.0006	-
				M	UR-M2	early*	18832	0.707208	0.000013	-	228	0.0044	-	-
SF	8	CvG_1	ca. 3300**	Indet.	LL-M3	late*	18833	0.707329	0.000009	0.0002	193	0.0052	0.0007	[58]
				Indet.	LR-M2	early*	18834	0.707136	0.000009	-	220	0.0045	-	-
		CvG_2	ca. 3300**	F	LL-M2	early*	18835	0.707237	0.000009	-	210	0.0048	-	[58]
	7	AgC_1	350 ± 64	M	UR-M3	late*	18839	0.706782	0.000010	0.0001	251	0.0040	0.0003	[46]
				M	UL-PM1	early*	18840	0.706854	0.000009	-	232	0.0043	-	-
	5	PA_1	340 ± 40	M	UR-M3	late*	18842	0.705603	0.000011	0.0006	114	0.0088	0.0017	[51]
				M	LR-M2	early	18843	0.706155	0.000014	-	141	0.0071	-	-
	6	Val_1		F	UL-M3	late*	18845	0.707367	0.000012	0.0018	276	0.0036	0.0042	-
				F	UL-M2	early	18846	0.709156	0.000012	-	128	0.0078	-	-
	4	Chi_1	84 ± 28	M	LR-M3	late*	18848	0.706067	0.000009	0.0002	155	0.0064	0.0004	[51]
				M	LR-PM2	early*	18849	0.706279	0.000011	-	146	0.0068	-	-
	3	Coli_1		Indet.	UL-M3	late*	18850	0.705962	0.000010	0.0000	173	0.0058	0.0003	-
				Indet.	LL-M2	early*	18851	0.706003	0.000014	-	181	0.0055	-	-
	2	MatJa_1		Indet.	LR-M3	late*	18852	0.707872	0.000009	0.0000	186	0.0054	0.0011	-
				Indet.	LR-PM1	early*	18853	0.707944	0.000011	-	153	0.0065	-	-
	1	Co_1		F	LL-M3	late*	18854	0.706140	0.000012	0.0006	142	0.0070	0.0013	-
				F	LL-M2	early*	18855	0.706663	0.000011	-	121	0.0083	-	-

Site refers to locations on Fig 1.

^a^U/L (Upper/Lower) L/R (Left/Right)—PM1/PM2/M1/M2/M3 (first and second premolar/first, second and third molar).

^b^Early crown mineralization ca. 2.5–8 years old and late ca. 13.5–15 years old [54].

*individuals with unidentified drinking water source [26].

**Chronology by direct association with human and mortuary structure remains of 3314 ± 51 and 3264 ± 38 years BP, respectively.

### Sampling and laboratory procedures

We analyzed the strontium isotopes and strontium concentration in the enamel of 54 permanent teeth from 33 individuals of both sexes (males = 14; females = 13 and indet. = 6) from the Negro River and the Somuncurá Foothills (NR = 23; SF = 10). Following AlQahtani et al. [54] dental development scheme, two teeth with different crown mineralization periods were sampled per individual (mostly M2 and M3, respectively) (Table 1).Samples were prepared and analyzed at the Departments of Archaeology and Geological Sciences of the University of Cape Town (UCT). Around 20 mg of enamel chunk was removed from the crown following the root axis and abraded -inner and outer layer- with a Dremel 3000 drill, with diamond heads washed with ethanol and ultrasonicated in MilliQ water to avoid cross-contamination [59]. Once the enamel chunk was mechanically cleaned, it was washed and ultrasonicated with MilliQ water for 20 min. It was then digested with 2 mL of 65% HNO_3_ in a closed Teflon beaker on a hotplate at 140°C for an hour. The samples were dried, re-digested in 1.7 mL of 2 M HNO_3_ and quantitatively split by weight into a 1.5-ml-fraction for strontium separation chemistry and a 0.2-ml-fraction for strontium element abundance analysis. The 0.2 ml fraction was analysed by quadrupole-ICP-MS for strontium elemental abundance using a Thermo Series II and a calibration curve constructed using artificial concentration standards. The elemental strontium in the 1.5 ml fraction was isolated with 200 μl of Triskem Sr.Spec resin following well-established routines [60]. The separated strontium fraction for each sample was dried down, dissolved in 2 mL 0.2% HNO_3_ and diluted to 200 ppb Sr concentrations for isotope analysis. Isotope ratios were measured using a Nu Instruments NuPlasma HR multicollector inductively-coupled-plasma mass spectrometer (MC-ICP-MS). Sample analyses were referenced to bracketing analyses of NIST SRM987 (^87^Sr/^86^Sr = 0.710255). Instrumental mass fractionation was corrected using the exponential mass bias law and a true ^86^Sr/^88^Sr value of 0.1194. Results for repeat analyses of an in-house carbonate standard processed and measured with the samples (^87^Sr/^86^Sr = 0.708925; 2σ = 0.000035; n = 33) agree with long-term results in this facility (^87^Sr/^86^Sr = 0.708911; 2σ = 0.000039; n = 545).

### Statistical analysis

The analysis was organized in two levels -population and individual- under a significance of *a* = 0.05 in R4.1.1 [61]. To perform a population level assessment, the human teeth ^87^Sr/^86^Sr data was described via Tukey’s IQR boxplot method and, complementary, kernel density estimation (package MASS::kde2d) by location and by sex. A generalized linear mixed-effect model was applied to evaluate the differences of the ^87^Sr/^86^Sr means grouping by different factors (package lmerTest, [62]). This kind of approach is particularly suitable for small, unbalanced and longitudinal datasets [63]. The model was built setting “^87^Sr/^86^Sr” as the dependent variable, and “Location”, “Sex” and “Mineralization” as fixed-effect factors with two levels each. The “Individual” was used as a random-effect factor to explicitly account for the non-independence among observations (i.e., some individuals have two isotope measurements generating pseudoreplication) [63]. This election is important since the violation of the independence assumption have massive effects on the type I error [64]. The Satterthwaite’s method was implemented for approximating degrees of freedom for the *F* tests and, consequently, more accurate *p*-values [62]. The residuals of the model were inspected to look for any deviation from normality and homoscedasticity that might obscure the results.

**Fig 2:**
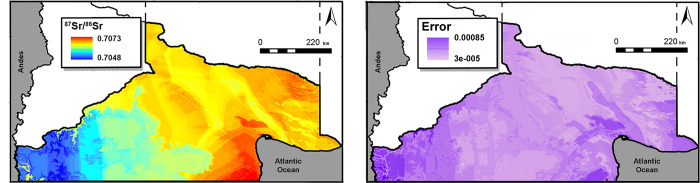
Bioavailable ^87^Sr/^86^Sr and uncertainty isoscapes. The maps represent the study area from which the bioarchaeological samples come from. Base map sourced from the R software package rworldmap (https://journal.r-project.org/archive/2011/RJ-2011-006/index.html).

The individual level assessment was performed exploring the ^87^Sr/^86^Sr isotope data against Sr concentrations in scatterplots (Table 1), where the offsets of paired early and late mineralization teeth were studied as absolute values and noted as Δ^87^Sr/^86^SrE-L and Δ1/[Sr]E-L for Sr isotope ratios and concentrations, respectively. Hierarchical Cluster analyses (ward method) were performed to organize the data into clusters based on the isotope and concentration similarities among individuals. To evaluate the most likely location that individuals exploited during the time of enamel formation, we implemented a continuous-surface probabilistic assignment approach using the package assignR [65]. We compared observed ^87^Sr/^86^Sr ratio in enamel with the bioavailable ^87^Sr/^86^Sr isoscape and its associated uncertainty (Fig 2); the function pdRaster creates a probability-of-provenance map where the sum of all cells on that map is 1.

## Results

### Distribution-based population assessment

**Fig 3:**
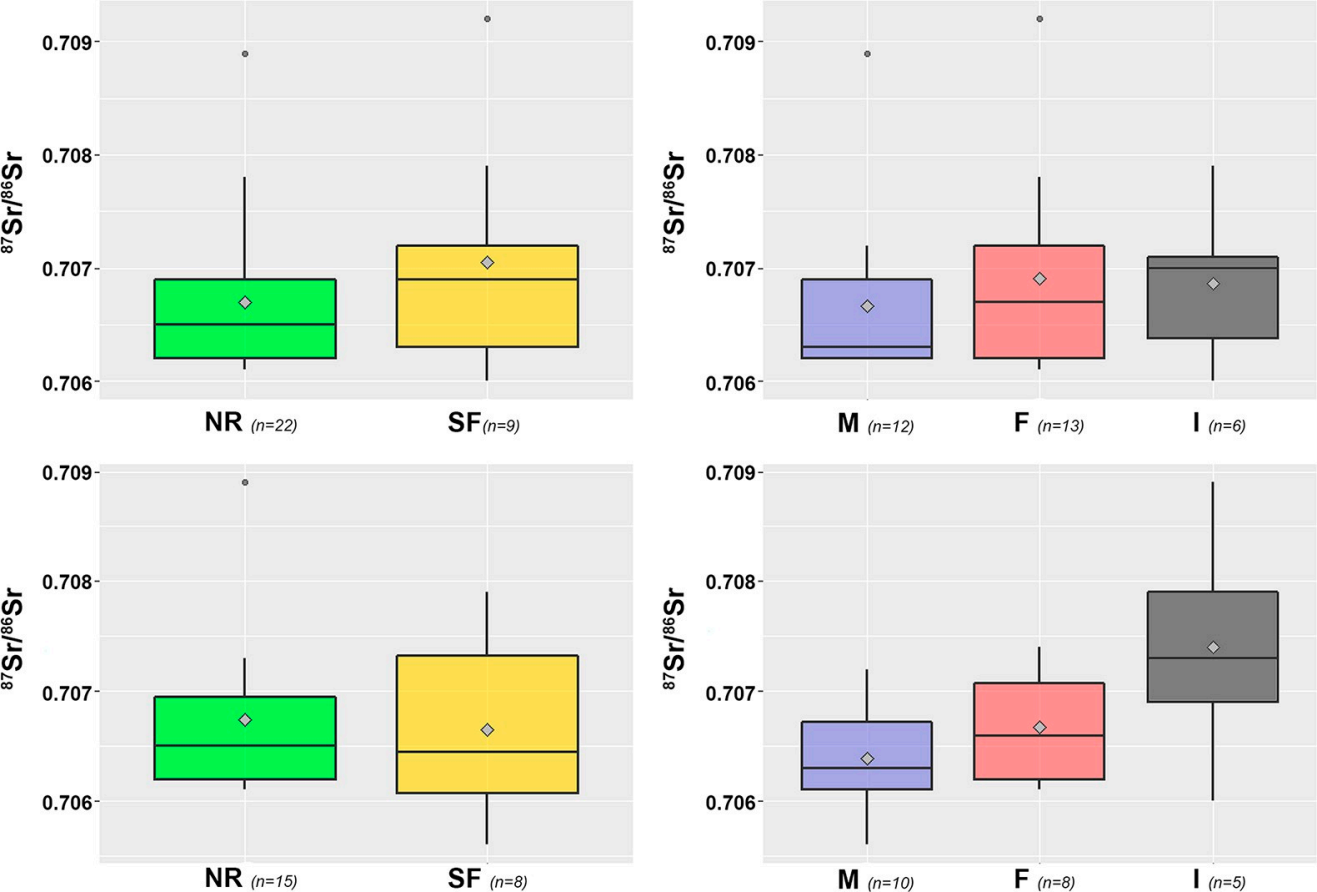
Dispersion of ^87^Sr/^86^Sr values. By location and sex from early (top row) and late (bottom row) mineralization teeth. NR: Negro River, SF: Somuncurá Foothills, M: male, F: female, I: indet. Grey diamonds represent the means.

Elemental and isotopic data from the enamel samples are listed in Table 1. Sr concentrations ([Sr]) and Sr isotope ratios (^87^Sr/^86^Sr) of the whole dataset (N = 54) range from 114.2 to 413.3 and 0.7056 to 0.7092 with median values of 202.1 and 0.7066, and mean values of 207.1 (± 59.8) and 0.7068 (± 0.0008) (0.7066 ± 0.0005, excluding outliers), respectively. When the ^87^Sr/^86^Sr dataset is split according to mineralization (Early and Late), similar patterns in the distributions arise in terms of location and sex (Fig 3). The interquartile ranges of the ^87^Sr/^86^Sr by location (Negro River -NR- and Somuncurá Foothills -SF-) are almost identical between teeth (Early NR = 0.7062–0.7069 vs Late NR = 0.7062–0.7070; Early SF = 0.7063–0.7072 vs Late SF = 0.7061–0.7073). Regardless of the teeth, the distributions of SF tend to overlap and show higher dispersion than those from NR (S1 Table). The kernel density plots illustrate the shapes of these distributions, showing that both Early and Late NR curves share a peak around 0.7061–0.7062 with the difference of a smoother descent in the latter. The SF curves share a smooth top around 0.7060 and the Late also displays a shoulder at ~0.7073 (S1 Fig).

The interquartile ranges by sex are similar between teeth (Early Male = 0.7062–0.7069 vs Late Male = 0.7061–0.7067; Early Female = 0.7062–0.7072 vs Late Female = 0.7062–0.7071) and, regardless of the teeth, they overlap, being female slightly wider. The interquartile range of indet. sex tends to show higher ^87^Sr/^86^Sr (0.7069–0.7079) and more dispersion from the mean (0.7070 ± 0.000922) for the late mineralization than for the early mineralization (0.7064–0.7071; 0.7068 ± 0.000787) (S1 Table). The kernels show that both early and late mineralization teeth in males have peaks at ~0.7061. The female curves are different between teeth, with the early mineralization teeth showing a bell-shaped curve with a peak at 0.7066, while the late mineralization teeth display both a moderate peak at ~0.7061 and a distinct shoulder around 0.7070. The indet. sex curves are both nearly bell-shaped, the early mineralization teeth showing a peak and shoulder at ~0.7071 and ~0.7061, respectively; and the late mineralization teeth displaying a smooth top around 0.7076 (S1 Fig). Three outliers with values of 0.7089 (SJ_Ñ-early, SJ_GQ-late) and 0.7092 (Val_1-early) were detected by the boxplots and represented as small curves set apart from the rest of the distributions by the kernels (Fig 3 and S2 Fig). The results of the linear mixed-effect model, excluding outliers and individuals of indet. sex, indicate that there are no statistically significant differences between locations (*F*_1,21.67_ = 0.1047, *p* = 0.7493), sexes (*F*_1,21.7_ = 1.8174, *p* = 0.1915) and time of teeth development (*F*_1,15.65_ = 3.3433, *p* = 0.0866). These results were validated by checking that the residuals of the model do not deviate from normality or homoscedasticity (S2 Fig).

### Baseline-based individual assessment

**Fig 4:**
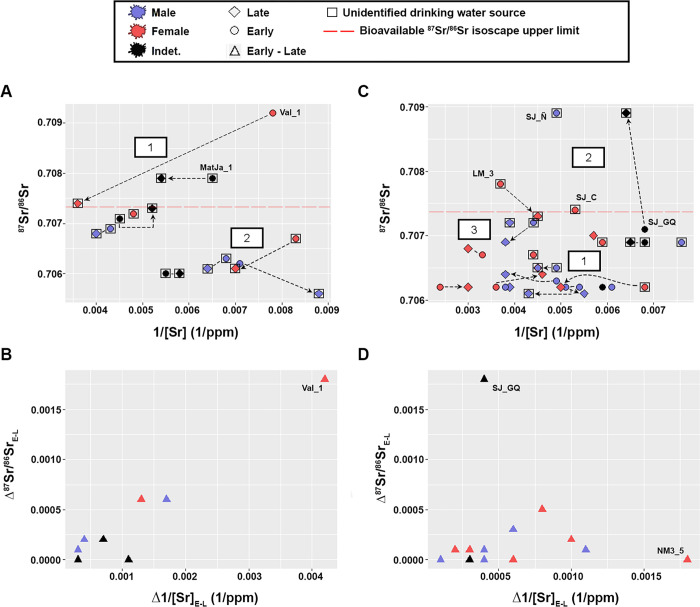
Strontium isotope ratios (^87^Sr/^86^Sr) vs strontium concentrations (1/[Sr]). Somuncurá Foothills -SF- (A) and Negro River -NR- (C). Early-late mineralization teeth paired samples are linked by arrows. Empty squares enclose individuals with unidentified drinking water sources (Table 1). Numbered boxes illustrate the relative locations of the main clusters suggested by the Hierarchical Cluster analysis (see the details of each cluster individual-by-individual in S3 Fig). The absolute values of the offsets of ^87^Sr/^86^Sr and 1/[Sr] between early-late paired enamel samples are from SF (B) and NR (D). Labelled individuals are mentioned in the text.

The ^87^Sr/^86^Sr and [Sr] from SF suggest two clusters (aggl. coef. = 0.9, S3A Fig), one of them displaying higher values than the other one (Fig 4A). Two individuals surpass the upper limit of the bioavailable ^87^Sr/^86^Sr isoscape defined for the study area during childhood and/or adolescence/early adulthood (Val_1-early = 0.7092; MatJa_1-early, late = 0.7079). Our results also show that the individuals from SF are tightly clustered around small offsets (≤ 0.0006 and ≤ 0.0017 1/ppm, respectively), being the only exception Val_1, who displays a remarkable change from a high Sr value and low concentration in childhood to a lower Sr value and higher concentration in adolescence/early adulthood (Fig 4B).

The values from NR show a cloudy pattern that might be organized into three clusters (aggl. coef. = 0.83, S3B Fig), one of them around 0.706 with [Sr] as its main axis of variation, and the other two around 0.707 with less variation in [Sr] (Fig 4C). The scatter plot also shows three outliers that exceed upper limit of the bioavailable ^87^Sr/^86^Sr isoscape at least in one moment of their lives: SJ_GQ-late (0.7089, 0.0064 1/ppm), SJ_Ñ-early (0.7089, 0.0049 1/ppm) and LM_3-early (0.7078, 0.0037 1/ppm) (Fig 4C). Similar to SF, the overall NR maintains the small strontium offset (≤ 0.0005) with one exception (SJ_GQ: 0.0018, 0.0004 1/ppm), but the sample is more scattered along the concentration axis, continuously ranging from 0.0001 to 0.0011 1/ppm, with one extreme value registered by NM3_5 (0.0018 1/ppm) (Fig 4D).

#### Probabilistic geographic assignment

**Fig 5:**
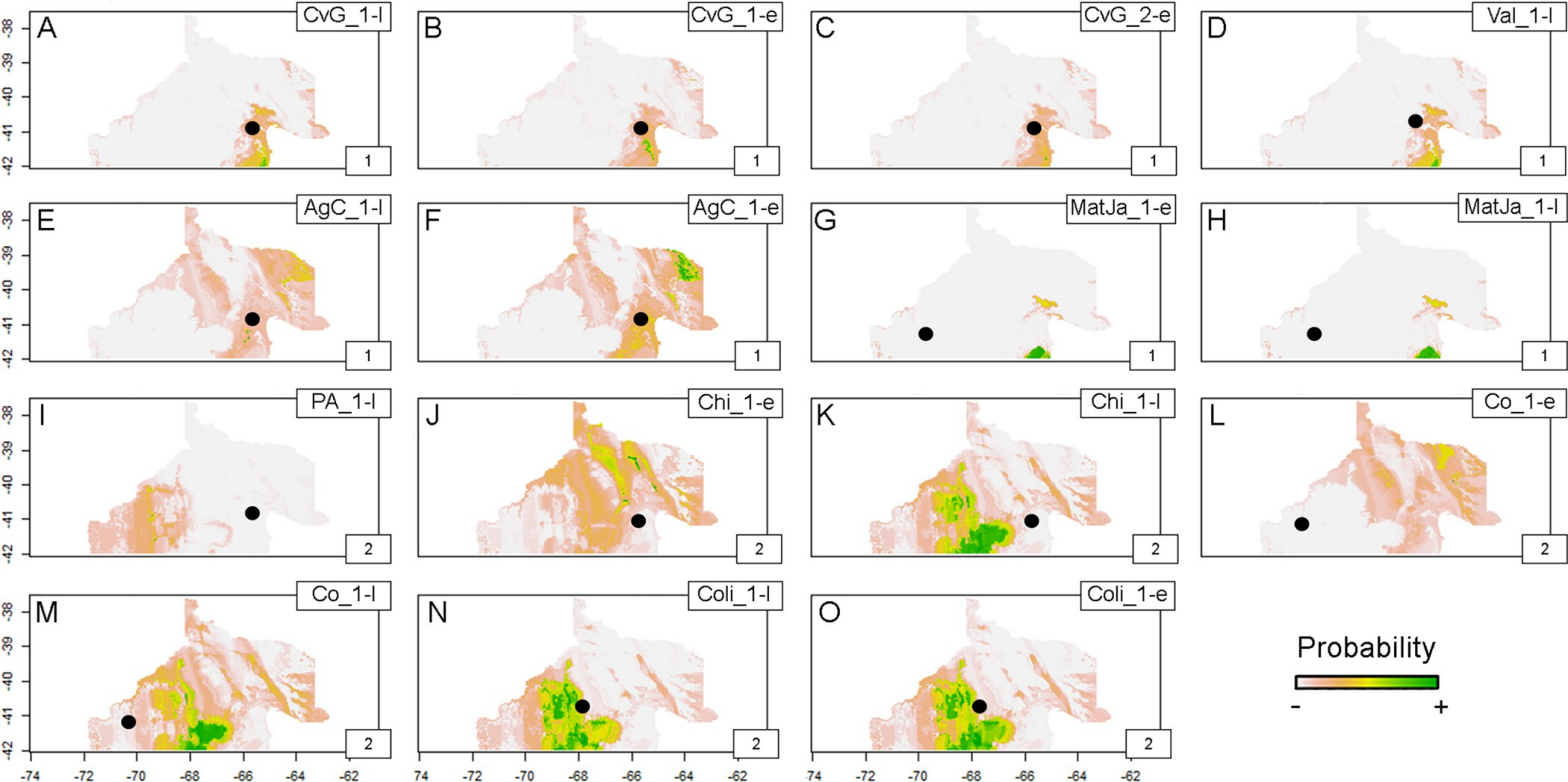
Probabilistic geographic assignment maps estimated using human enamel ^87^Sr/^86^Sr from SF sample. Black dots signal burial location. Numbered boxes on the bottom-right corner of each map refer to the clusters (see Fig 4A and S3A Fig). Base map sourced from the R software package rworldmap (https://journal.r-project.org/archive/2011/RJ-2011-006/index.html).

The probability maps for the SF sample show that one of the aforementioned clusters -mostly eastern burials- contains individuals that are relatively local with potential region-of-origin in the nearby coast or, in some cases, in northern river valleys (Fig 5A–5F). Burials from the western side of the Foothill show the highest probability of origin in nearby coastal areas (Fig 5G and 5H). The other cluster from the SF sample also has burials from both east and west of the Foothill. Those buried at the east have high probabilities over western Foothills (Fig 5I), northern valleys (Fig 5J), and the Somuncurá Plateau (Fig 5K). Individuals from the west show higher probabilities around the northeastern (Fig 5L) and the Somuncurá and El Cuy Plateaus (Fig 5M–5O).

**Fig 6:**
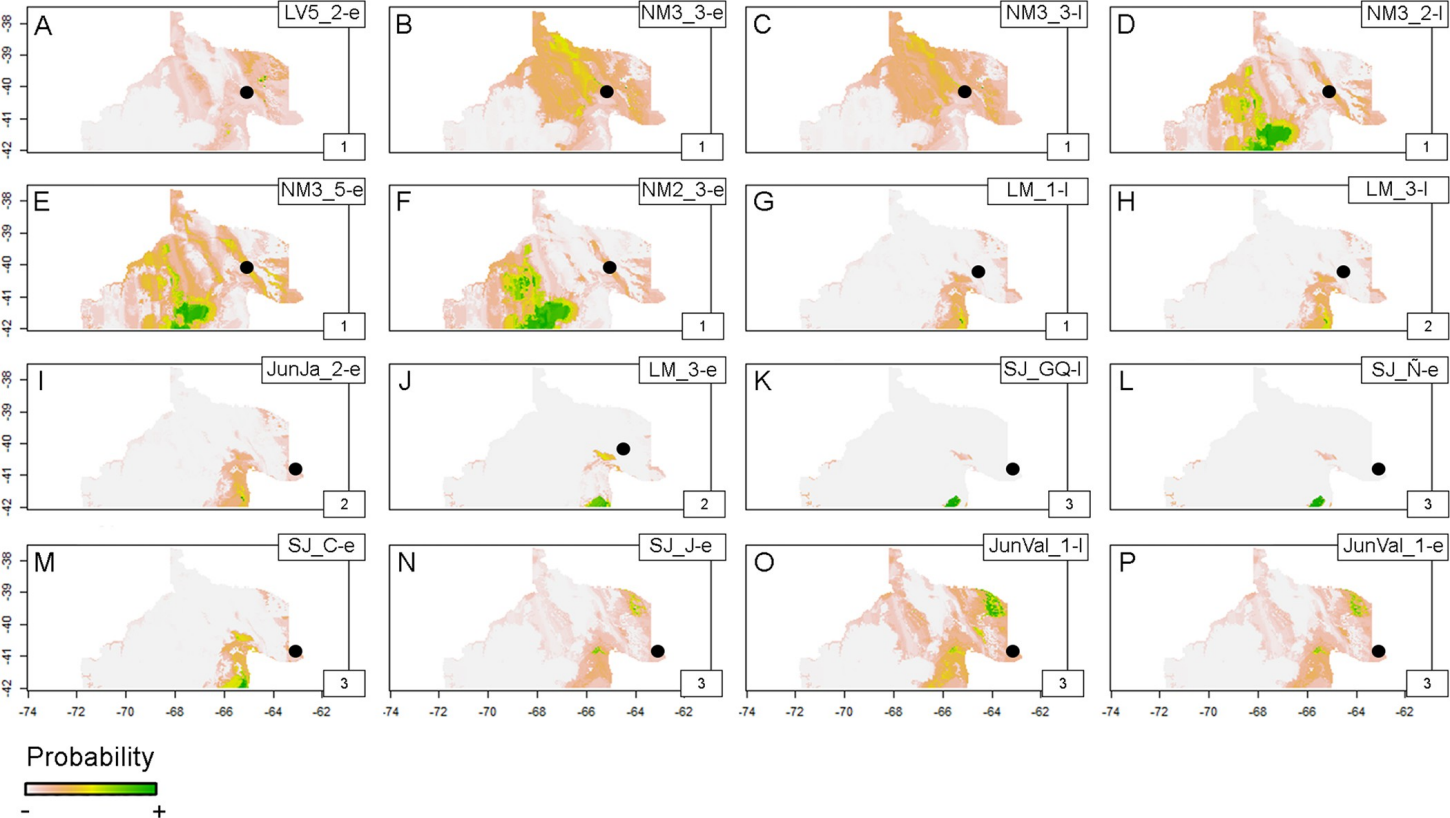
Probabilistic geographic assignment maps estimated using human enamel ^87^Sr/^86^Sr from NR sample. Black dots signal burial location. Numbered boxes on the bottom-right corner of each map refer to the clusters (see Fig 4C and S3B Fig). Base map sourced from the R software package rworldmap (https://journal.r-project.org/archive/2011/RJ-2011-006/index.html).

For the NR sample, its three clusters comprise individuals with high probability of origin in the northern areas and/or the coast around western Foothills (Fig 6G–6P). The other individuals are mostly compatible with a local exploitation of Negro and Colorado valleys (Fig 6A–6C) and with the Somuncurá Plateau (Fig 6D–6F).

## Discussion

### General mobility trends

Most of the individuals have ^87^Sr/^86^Sr values around ~0.706–0.707 (Figs 3, 4A and 4C), and show limited differences based on location, sex and mineralization. The study of the offsets also shows relative stability of the ^87^Sr/^86^Sr for most of the individuals as they advance in age (Fig 4B and 4D). These isotope values are compatible with the bioavailable ^87^Sr/^86^Sr baseline predicted for the study area (Fig 2), reinforcing its suitability to evaluate past human mobility (see discussion in [42]). These results also indicate that most individuals buried in the study area most likely dwelled or predominantly exploited this region during the time of tissue development. While the southwest -location with the lowest ^87^Sr/^86^Sr- does not seem to have been intensively exploited, there is an overall sustained use of northern Negro and Colorado valleys and Somuncurá/coast -locations with higher ^87^Sr/^86^Sr- (Fig 2). Regardless of the ecological and/or social motivations, the recurrent use of specific locations is expected in Patagonia. The archaeological and bioarchaeological records show that Late Holocene hunter-gatherers often re-used the same locations (“persistent places”, *sensu* [66]) for settlement and inhumation through time (see examples in [49, 67, 68]). As long as critical resources are available, the daily circulation around the same area for an indefinite amount of time is one possible landscape exploitation strategy among hunter-gatherer groups [15, 69–71].

### Individual mobility and flow of exotic items

**Fig 7:**
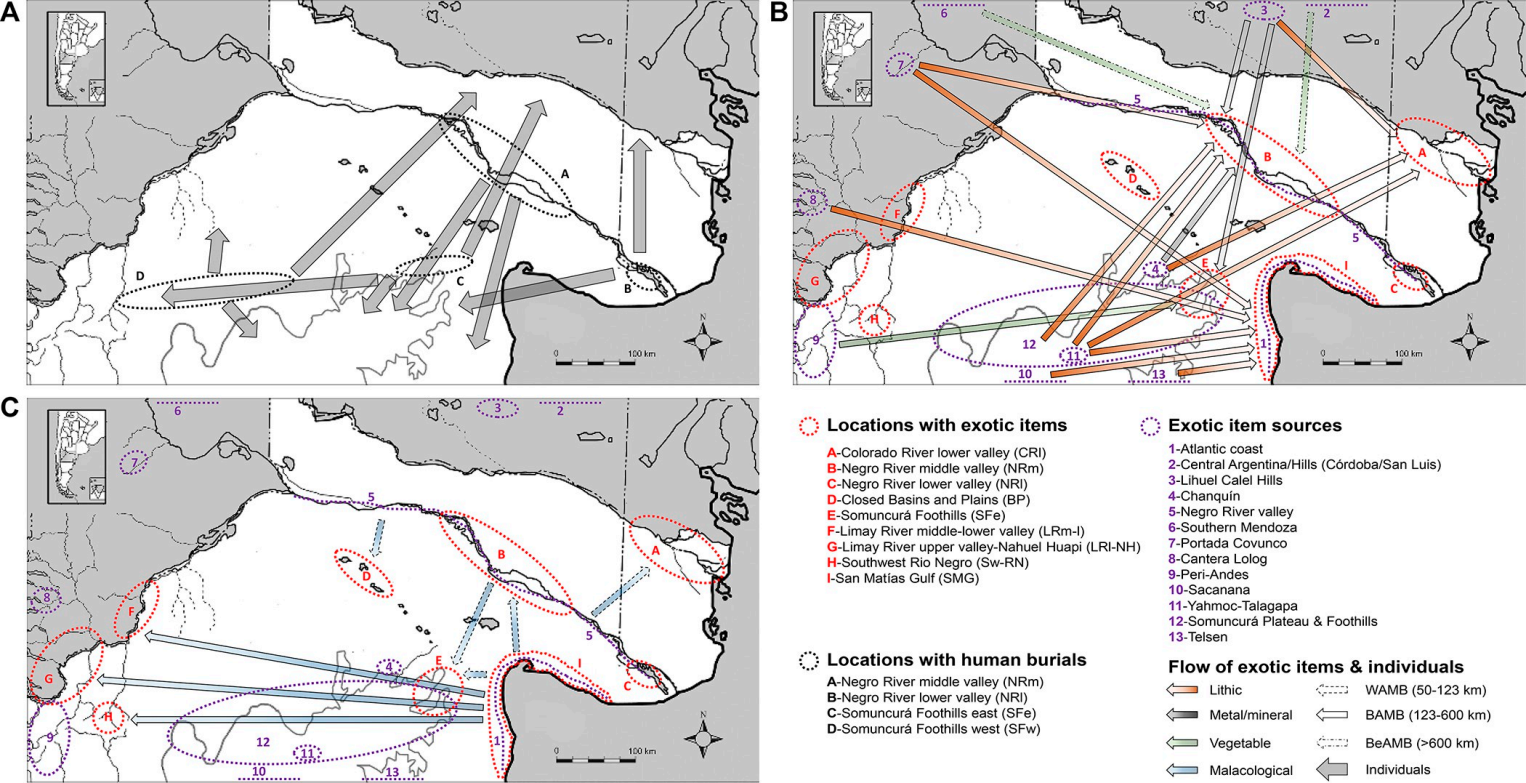
Human mobility and flow of exotic items within the study area during the Late Holocene. A) Potential axes of extraordinary mobility based on the assignments from Figs 5 and 6. Arrows represent more than one individual with similar assignment; B) Flow of lithics, metals/minerals, and vegetables; C) Flow of malacological material. WAMB: exotic within adjacent maximal band; BAMB: exotic between adjacent maximal band; BeAMB: exotic beyond adjacent maximal band. Items from colonial period are not included. For cases with multiple potential sources, only the closest ones are included (see details in S2 Table). Adapted from A. Serna Unpublished PhD thesis under a CC BY license, with permission of the author, original copyright 2018.

The assignments of the SF sample suggest sustained local exploitation of the eastern Foothills including the adjacent coast for some individuals buried in the East (Fig 5A–5F). For other individuals, whose burial locations vary across the Foothills, their strontium isotope ratios point towards different sectors of this southern area (Fig 5G–5O). The NR sample highlights two general patterns of potential exploitation: -local- Negro and Colorado valleys (Fig 6A–6C), and -southern- Somuncurá Plateau (Fig 6D–6F) and the coast around the eastern Foothills (Fig 6G–6P). The surfaces of high probability are broad, which limit a more precise interpretation, but they also represent highly unproductive “drylands” such as the *travesía* and the Negro-Colorado interfluve (see Fig 1) [26, 46, 47]. Once dismissed these landscapes of unlikely systematic exploitation, some patterns may arise. Aside from cases of local exploitation, high probabilities also suggest virtual connections between burial locations and distant exploitation areas (see Fig 7A). These connections, that describe extraordinary mobility, might be summarized and hypothesized as three general axes: 1) east-west of the Somuncurá Foothills, 2) Negro-Colorado River valleys and 3) Negro River-Somuncurá/coast. These results are compatible with our previous knowledge based on oxygen (δ^18^O), which pointed to the first two axes of mobility [26], but they also make evident the potential connection between the Negro River and Somuncurá. Although these axes of mobility are hypothetical, the environmental constraints imposed by the water availability and the dangers of crossing the *travesía* [48] make them highly plausible. On the one hand, the natural corridor created by the drainage system of the Somuncurá Foothills would have allowed a fluid east-west transit between the coast and the steppes/foothills [26, 46, 47]. On the other hand, the ecological conditions of the coast (e.g., high bio-productivity, availability of raw materials, springs) [72] would have been favorable enough to guarantee a safe north-south transit between the Negro River and Somuncurá.

Similarly to the axes of extraordinary mobility above mentioned (Fig 7A), the flow of exotic items (S2 Table) might be summarized as two main axes of flowing: coast and peri-Andes via South (southern east-west connection), and Foothills/coast and Negro River via East (eastern north-south connection) (see Fig 7B and 7C). Overall and considering the less likely systematic exploitation of the *travesía*, both human mobility and item flowing axes overlap around the eastern Somuncurá/coast. This convergence indicates that this location could have been relevant for articulating people and items (e.g., as an intermediate location for trading). At least in colonial times, several roads converged in this location that was known as one of the main nodes connecting southern and northern Patagonia [48]. The fact that the potential axes of extraordinary mobility overlap with the predominant flows of exotic items could indicate that some kind of small-scale socially driven mobility might have operated. In terms of Whallon [14], this mechanism of interaction is known as “non-utilitarian” mobility and, without being related to subsistence tasks, it seeks to expand and reinforce social ties through the exchange of items and information, as well as to fulfil social expectations.

Considering the non-utilitarian mobility as a potential mechanism of interaction, it is also possible to state that the objects might have been the ones circulating -rather than large groups of people moving and settling-. Aside from the fact that a few individuals presented isotope values compatible with extraordinary patterns of mobility, the distances between the sources and the locations of the exotic items are also suggestive. According to Whallon’s geographic model ([14]: 266), the vast majority of flows in the study area would exceed the limits (ca. >123 km) of a maximal band -i.e., smaller bands linked in mutual contact-, and they would occur between adjacent maximal bands (*n* = 46, 79%) and beyond (*n* = 2, 3%) (S2 Table). Three basic procurement mechanisms might be considered to discuss these flows: embedded procurement within regular regional mobility, direct procurement by specific trips to the sources, and indirect procurement through exchange networks [73]. According to ethnographic data on hunter-gatherers [74], daily forays are usually short (ca. <10 km from the camp), and even those of large distances with overnights do not exceed 100 km (88 km, 20 km/day). Although we do not discard the influence of the direct procurement by specific trips, our results suggest that part of the exotic item circulation might have occurred through exchange networks fostered by small parties of individuals that visited acquaintances, carried exotic materials and resided in different -distant- locations within North Patagonia.

Individuals performing some kind of non-utilitarian mobility carrying and exchanging items between social units are aligned with ethnographic inferences, which indicate that the annual inter-band interaction rate among hunter-gatherers is high and that the ritual relationship is a strong driver of this interaction [16]. Hence, it is expected that the archaeological record from neighbouring regions shows potential evidence of this kind of process. Located in the Dry Pampas -north of the study area-, the Late Holocene Chenque I cemetery has at least two individuals potentially from the distant Andean region, which supports long-distance networks among groups from Central Argentina and the Andes [75]. Although it occurred during colonial (equestrian) times, the Rawson site in the lower valley of the Chubut River -south of the study area- displays one of the most striking pieces of evidence of exotic items in Patagonia with the finding of a bronze ceremonial axe potentially from the Argentinian Northwest [76]. Regardless of the items either flowing within or between different social units, it is probable that the exchange -in any way- has played a crucial role in the exotic item circulation in the study area and beyond. While the variety of the specific interaction mechanisms deserves attention, this work evidences the relevance of small-scale socially driven mobility for the materialization and maintenance of the Patagonian hunter-gatherer exchange networks and promotes reflection on how mobile these groups were and how they interacted with each other.

## Conclusions

In this paper, we have explored the hunter-gatherer mobility patterns and their potential connections with the flow of exotic items in North Patagonia during the Late Holocene. The analysis of strontium isotopes fits with previous findings about mobility and suggests a predominant exploitation of a dietary catchment within the study area for most of the individuals. Nevertheless, the isotope values of some individuals suggest extraordinary mobility that could be compatible with the main flows of exotic items in the study area. As in colonial times, the eastern sector of the Somuncurá Foothills must have been a central point of convergence of people and items from different locations from around the broader region.

Without neglecting the multipurpose nature of mobility, we argue that small-scale non-utilitarian mobility (*sensu* [14]) might have operated in the study area and that the exotic items were the ones circulating rather than large groups of people moving and settling. Supported by ethnographic and archaeological evidence, most of the observed flows of exotic items might have occurred through exchange networks fostered by individuals with extraordinary mobility that visited people, carried exotic materials, and resided occasionally in different locations. While our results highlight the relevance of small-scale human circulation as a foster for the Patagonian exchange networks, future follow-up work is needed to better understand the mechanisms and the role of specific agents and locations in this process.

## Supporting information

S1 Fig Kernel density estimation by location and sex. Early (top row) and Late (bottom row) mineralization teeth.
(TIF)

S2 Fig Contrast of the assumptions for the linear mixed-effect model. Histogram with normality test (left) and residual plot (right).
(TIF)

S3 Fig Hierarchical Cluster analyses for SF (A) and NR (B) samples. * signals unidentified drinking water individuals.
(TIF)

S1 Table Summary of the ^87^Sr/^86^Sr values from Fig 3. (DOCX)

S2 Table Flow of exotic items within the study area during the Late Holocene. Materials from the colonial period are not included (see also Fig 7).
(DOCX)

## References

[1] Bettinger RL. Aboriginal Human Ecology in Owens Valley: Prehistoric Change in the Great Basin. Am Antiq. 1977;42: 3–17. doi: 10.2307/279458

[2] Binford LR. Nunamiut Ethnoarchaeology. New York: Academic Press; 1978.

[3] Clark G. The Invasion Hypothesis in British Archaeology. Antiquity. 1966;40: 172–189. doi: 10.1017/S0003598X00032488

[4] Chapman J, Hamerow H. Migrations and Invasions in Archaeological Explanation (British Archaeological Reports International Series 664). Oxford: Archaeopress; 1997.

[5] Shaffer Lichtenstein. South Asian archaeology and the myth of Indo-Aryan invasions. The Indo-Aryan Controversy. 2004.

[6] Hakenbeck S. Migration in archaeology: Are we nearly there yet. Archaeological Review from Cambridge. 2008;23: 9–26.

[7] Cabana GS, Clark JJ. Rethinking anthropological perspectives on migration. Gainesville: University Press of Florida; 2011.

[8] Knudson KJ, Goldstein PS, Dahlstedt A, Somerville A, Schoeninger MJ. Paleomobility in the Tiwanaku diaspora: biogeochemical analyses at Rio Muerto, Moquegua, Peru. Am J Phys Anthropol. 2014;155: 405–421. doi: 10.1002/ajpa.22584 25066931

[9] van Dommelen P. Moving On: Archaeological Perspectives on Mobility and Migration. World Archaeol. 2014;46: 477–483. doi: 10.1080/00438243.2014.933359

[10] Cameron CM. How people moved among ancient societies: Broadening the view. Am Anthropol. 2013;115: 218–231. doi: 10.1111/aman.12005

[11] Kelly RL. Hunter-Gatherer Mobility Strategies. J Anthropol Res. 1983;39: 277–306. doi: 10.1086/jar.39.3.3629672

[12] Binford LR. Willow smoke and dogs’ tails: hunter-gatherer settlement systems and archaeological site formation. Am Antiq. 1980;45: 4–20.

[13] Grove M. Hunter–gatherer movement patterns: Causes and constraints. Journal of Anthropological Archaeology. 2009;28: 222–233. doi: 10.1016/j.jaa.2009.01.003

[14] Whallon R. Social networks and information: Non-“utilitarian” mobility among hunter-gatherers. Journal of Anthropological Archaeology. 2006;25: 259–270. doi: 10.1016/j.jaa.2005.11.004

[15] Kelly RL. The Lifeways of Hunter-Gatherers: The Foraging Spectrum. Cambridge University Press; 2013.

[16] Hill KR, Wood BM, Baggio J, Hurtado AM, Boyd RT. Hunter-gatherer inter-band interaction rates: implications for cumulative culture. PLoS One. 2014;9: e102806. doi: 10.1371/journal.pone.0102806 25047714 PMC4105570

[17] Wobst HM. Stylistic behavior and information exchange. In: Cleland CE, editor. Papers for the Director: Research Essays in Honor of James B Griffin. Ann Arbor: University of Michigan Museum of Anthropology; 1977. pp. 317–342.

[18] Knappett C. Meaning in miniature: semiotic networks in material culture. Excavating the mind. 2012; 87–109.

[19] Robb J. 12 what do things want? Object design as a middle range theory of material culture. Archeol pap Am Anthropol Assoc. 2015;26: 166–180. doi: 10.1111/apaa.12069

[20] Brettell R, Evans J, Marzinzik S, Lamb A, Montgomery J. ‘impious easterners’: Can oxygen and strontium isotopes serve as indicators of provenance in early medieval European cemetery populations? Eur J Archaeol. 2012;15: 117–145. doi: 10.1179/1461957112y.0000000001

[21] Gregoricka LA. Moving Forward: A Bioarchaeology of Mobility and Migration. Journal of Archaeological Research. 2021;29: 581–635. doi: 10.1007/s10814-020-09155-9

[22] Gómez Otero J. Dieta, uso del espacio y evolución en poblaciones cazadoras-recolectoras de la costa centroseptentrional de Patagonia durante el Holoceno medio y tardío. Phd, Universidad de Buenos Aires, Argentina. 2006. Available: http://repositorio.filo.uba.ar/handle/filodigital/1274.

[23] Prates L. Los indígenas del río Negro: un enfoque arqueológico. Sociedad Argentina de Antropología; 2008.

[24] Martínez G, editor. Arqueología de cazadores-recolectores del curso inferior del Río Colorado: provincia de Buenos Aires, Argentina: aportes al conocimiento de las ocupaciones humanas Pampeano-Patagónicas. Olavarría: INCUAPA-CONICET y UNICEN; 2017.

[25] Mange E. Investigaciones arqueológicas en la margen sur del valle medio-superior del río Negro (pcia de Río Negro). Phd, Universidad Nacional de La Plata, Argentina. 2019. Available: http://sedici.unlp.edu.ar/handle/10915/80578.

[26] Serna A, Salazar-García DC, Valenzuela LO, Prates L. A tough travesía: Mobility constraints among late Holocene Patagonian hunter-gatherers through oxygen stable isotopes in enamel and water sources. Journal of Archaeological Science: Reports. 2020;33: 102484. doi: 10.1016/j.jasrep.2020.102484

[27] Scheinsohn V. Rock art information among hunter-gatherers in Northwest Patagonia: an assessment of broad-scale and territorial models. 73rd Annual Meeting of the Society for American Archaeology (SAA). researchgate.net; 2011. pp. 235–247.

[28] Acevedo A. Hachas grabadas, placas grabadas y comunicación visual suprarregional entre grupos cazadores-recolectores de finales del Holoceno tardío. Relaciones de la Sociedad Argentina de Antropología. 2015;XL: 589–620.

[29] Di Prado V. Prácticas alfareras prehispánicas y procesos de interacción social en el centro-este de Argentina durante el Holoceno tardío. Lat Am Antiq. 2018;29: 552–571. doi: 10.1017/laq.2018.28

[30] Carden N, Martínez G. Diseños Fragmentados: Circulación Social de Imágenes Sobre Huevos de Rheidae en Pampa y Norpatagonia. Bol Mus Chil Arte Precolomb. 2014;19: 55–75. doi: 10.4067/S0718-68942014000200004

[31] Serna A, Prates L, Flensborg G, Martínez G, Favier Dubois C, Perez SI. Does the shape make a difference? Evaluating the ethnic role of cranial modification in the Pampa-Patagonia region (Argentina) during the late Holocene. Archaeol Anthropol Sci. 2019;11: 2597–2610. doi: 10.1007/s12520-020-01010-8

[32] Martínez G, Santos Valero F, Flensborg G, Carden N, Stoessel L, Alcaraz AP, et al. Was There a Process of Regionalization in Northeastern Patagonia During the Late Holocene? J Isl Coast Archaeol. 2017;12: 95–114. doi: 10.1080/15564894.2016.1163756

[33] Saghessi D, Mange E. Artefactos picados y/o abradidos procedentes de sitios arqueológicos y colecciones del valle medio del río Negro (Rio Negro, Argentina). Revista Museo La Plata Suplemento Resúmenes. 2017;2: 1R–13R.

[34] Di Lorenzo M, Mange E, Hammond H, Prates L. El uso de moluscos marinos entre los grupos cazadores recolectores del interior norpatagónico en el Holoceno tardío (provincia de Río Negro, Argentina). Arqueología. 2022;28: 9926–9926. doi: 10.34096/arqueologia.t28.n1.9926

[35] Fernández M, Vitores M. Distribución de la cerámica arqueológica en la cuenca superior y media del río Limay. III Jornadas de Historia de la Patagonia. San Carlos de Bariloche: Universidad Nacional del Comahue; 2008.

[36] Schuster V. La organización tecnológica de la cerámica de cazadores-recolectores. Costa norte de la Provincia del Chubut (Patagonia Argentina). Relaciones de la Sociedad Argentina de Antropología. 2014;XXXIX: 203–231.

[37] Pérez AE, Erra G. Identificación de maiz de vasijas recuperadas de la Patagonia noroccidental argentina. Magallania. 2011;39: 309–316. doi: 10.4067/S0718-22442011000200022

[38] Prates L, Serna A, Mange E, López L, Romano V, Di Lorenzo M, et al. Ocupaciones residenciales y entierros humanos en negro muerto 3 (Valle del Río Negro, Norpatagonia). Magallania. 2019;47: 159–176. doi: 10.4067/S0718-22442019000100159

[39] Capo RC, Stewart BW, Chadwick OA. Strontium isotopes as tracers of ecosystem processes: theory and methods. Geoderma. 1998;82: 197–225. doi: 10.1016/S0016-7061(97)00102-X

[40] Bentley RA. Strontium Isotopes from the Earth to the Archaeological Skeleton: A Review. Journal of Archaeological Method and Theory. 2006;13: 135–187. doi: 10.1007/s10816-006-9009-x

[41] Bataille CP, von Holstein ICC, Laffoon JE, Willmes M, Liu X-M, Davies GR. A bioavailable strontium isoscape for Western Europe: A machine learning approach. PLoS One. 2018;13: e0197386. doi: 10.1371/journal.pone.0197386 29847595 PMC5976198

[42] Serna A, Prates L, Mange E, Salazar-García DC, Bataille CP. Implications for paleomobility studies of the effects of quaternary volcanism on bioavailable strontium: A test case in North Patagonia (Argentina). J Archaeol Sci. 2020;121: 105198. doi: 10.1016/j.jas.2020.105198

[43] Paruelo JM, Beltrán A, Jobbágy E, Sala OE, Golluscio RA. The climate of Patagonia: general patterns and controls on biotic processes. Asociación Argentina de Ecología Ecol Austral. 1998.

[44] Stern LA, Blisniuk PM. Stable isotope composition of precipitation across the southern Patagonian Andes. J Geophys Res D: Atmos. 2002;107: ACL 3-1-ACL 3–14. doi: 10.1029/2002JD002509

[45] Schäbitz F. Estudios polínicos del Cuaternario en las regiones áridas del sur de Argentina. Rev Mus Argent Cienc Nat. 2003;5: 291–299.

[46] Prates L, Mange E. Paisajes de tránsito y estaciones en las planicies y bajos del centro-este de Norpatagonia. Relaciones de la Sociedad Argentina de Antropología. 2016;XLI: 217–236.

[47] Serna A, Prates L, Valenzuela LO, Salazar-García DC. Back to the bases: Building a terrestrial water δ18O baseline for archaeological studies in North Patagonia (Argentina). Quat Int. 2020;548: 4–12. doi: 10.1016/j.quaint.2019.06.008

[48] Casamiquela R. Bosquejo de una etnología de la Provincia de Río Negro. Viedma: Fundación Ameghino; 1985.

[49] Prates L, Di Prado V. Sitios con entierros humanos y ocupaciones residenciales en la cuenca del Río Negro (Norpatagonia, Argentina): Diacronía y Multicausalidad. Lat Am Antiq. 2013;24: 451–466. doi: 10.7183/1045-6635.24.4.451

[50] Prates L, Ballejo F, Blasi A. Analysis of hair remains from a hunter-gatherer grave from Patagonia: Taxonomic identification and archaeological implications. Journal of Archaeological Science: Reports. 2016;8: 142–146. doi: 10.1016/j.jasrep.2016.05.064

[51] Serna A. Interacciones sociales en el noreste de Patagonia durante el Holoceno tardío: un enfoque bioarqueológico. Phd, Universidad Nacional de La Plata, Argentina. 2018. Available: http://sedici.unlp.edu.ar/handle/10915/68142.

[52] Serna A, Romano V. Rescates bioarqueológicos en el valle medio del río Negro (provincia de Río Negro): el potencial informativo del registro altamente perturbado. Rev argent antropol biol. 2018;20: 1–12. doi: 10.17139/raab.2018.0020.02.03

[53] Madrid PE, Barrientos G. La estuctura del registro arqueológico del sitio Laguna Tres Reyes I (provincia de Buenos Aires): nuevos datos para la interpretación del poblamiento humano en el sudeste de la región pampeana a inicios del Holoceno tardío. Relaciones de la Sociedad Argentina de Antropología. 2000;25: 179–206.

[54] AlQahtani SJ, Hector MP, Liversidge HM. Accuracy of dental age estimation charts: Schour and Massler, Ubelaker and the London Atlas. Am J Phys Anthropol. 2014;154: 70–78. doi: 10.1002/ajpa.22473 24470177

[55] Prates L, Di Prado V, Mange E, Serna A. Sitio Loma de los Muertos: Múltiples ocupaciones sobre un médano del este de Norpatagonia (Argentina). Magallania. 2010;38: 165–181. doi: 10.4067/S0718-22442010000100010

[56] Serna A, Prates L. Bioarqueología y cronología del sitio Negro Muerto 2 (Noreste de Patagonia). Magallania. 2012;40: 233–245. doi: 10.4067/S0718-22442012000200011

[57] Prates L, Luchsinger H, Scabuzzo C, Mansegosa D. Investigaciones arqueológicas en el sitio La Victoria 5 (Departamento de General Conesa, Río Negro). Intersecc antropol. 2011;12: 109–120.

[58] Carden N, Prates L. Pinturas rupestres en un espacio funerario: El caso del sitio Cueva Galpón (Noreste de Patagonia). Magallania. 2015;43: 117–136. doi: 10.4067/S0718-22442015000100008

[59] Budd P, Montgomery J, Barreiro B, Thomas RG. Differential diagenesis of strontium in archaeological human dental tissues. Appl Geochem. 2000;15: 687–694. doi: 10.1016/S0883-2927(99)00069-4

[60] Pin C, Briot D, Bassin C, Poitrasson F. Concomitant separation of strontium and samarium-neodymium for isotopic analysis in silicate samples, based on specific extraction chromatography. Anal Chim Acta. 1994;298: 209–217. doi: 10.1016/0003-2670(94)00274-6

[61] R Core Team. A Language and Environment for Statistical. R Foundation for Statistical Computing. Vienna, Austria. https://www.r-project.org. 2021.

[62] Kuznetsova A, Brockhoff PB, Christensen RHB. lmerTest package: tests in linear mixed effect models. J Stat Softw. 2017;82: 1–26. doi: 10.18637/jss.v082.i13

[63] Maxwell SE, Delaney HD, Kelley K. Designing experiments and analyzing data: A model comparison perspective (3rd edition). New York: Routledge; 2018.

[64] Winter B. Statistics for Linguists: An Introduction Using R. New York: Routledge; 2019.

[65] Ma C, Vander Zanden HB, Wunder MB, Bowen GJ. assignR: An r package for isotope‐based geographic assignment. Methods Ecol Evol. 2020;11: 996–1001. doi: 10.1111/2041-210x.13426

[66] Schlanger SH. Recognizing Persistent Places in Anasazi Settlement Systems. In: Rossignol J, Wandsnider L, editors. Space, Time, and Archaeological Landscapes. Boston, MA: Springer US; 1992. pp. 91–112. doi: 10.1007/978-1-4899-2450-6_5

[67] Martínez G, Flensborg G, Bayala PD. Human corpse manipulation and the body as symbol: A case study from the Eastern Pampa–Patagonia transition (Argentina) during the Final Late Holocene. J Anthropol Archaeol. 2012;31: 215–226. doi: 10.1016/j.jaa.2011.12.002

[68] Zilio L, Hammond H. A Persistent Place for Hunter-Gatherers During the Late Holocene: The Case of Burials in Pit on the Coast of Lángara Bay, Argentine Patagonia. J Isl Coast Archaeol. 2018;13: 438–449. doi: 10.1080/15564894.2017.1284962

[69] Pate FD. Stable carbon isotope assessment of hunter—Gatherermobility in prehistoric South Australia. J Archaeol Sci. 1995;22: 81–87. doi: 10.1016/S0305-4403(95)80164-2

[70] Pate FD. Bone Collagen Stable Nitrogen and Carbon Isotopes as Indicators of Past Human Diet and Landscape Use in Southeastern South Australia. Australian Archaeology. 1998; 23–29.

[71] Diet Sealy J., mobility, and settlement pattern among Holocene Hunter‐gatherers in southernmost Africa. Curr Anthropol. 2006;47: 569–595. doi: 10.1086/504163

[72] Favier Dubois CM, Borella F, Tykot R. Explorando tendencias en el uso humano del espacio y los recursos en el litoral rionegrino (Argentina) durante el Holoceno medio y tardío. In: Salemme M, Santiago F, Álvarez M, Piana E, Vázquez M, Mansur ME, editors. Arqueología de Patagonia: una mirada desde el último confín. Ushuaia: Editorial Utopías; 2009. pp. 985–997.

[73] Morrow CA, Jefferies RW. Trade or embedded procurement?: a test case from southern Illinois. In: Torrence R, editor. Time, Energy and Stone Tools. Cambridge: Cambridge University Press; 1989. pp. 27–33. Available: https://ci.nii.ac.jp/naid/10006683361/.

[74] Kelly RL. Obsidian in the Carson Desert. Perspectives on prehistoric trade and exchange in California and the Great Basin. In: Hughes R, editor. Perspectives on Prehistoric Trade and Exchange in the California and the Great Basin. Salt Lake City: University of Utah Press; 2011. pp. 189–200.

[75] Berón M, Di Biase A, Musaubach G, Páez F. Enclaves y espacios internodales en la dinámica de poblaciones en el Wall-Mapu: aportes desde la arqueología pampeana. Estud atacam. 2017; 253–272. doi: 10.4067/S0718-10432017005000008

[76] Gómez Otero J, Dahinten S. Bioarqueología de la costa centro-septentrional de Patagonia Argentina. In: Cruz I, Caracotche MS, editors. Arqueología de la costa patagónica Perspectivas para la conservación. Río Gallegos: Universidad Nacional de la Patagonia Austral; 2006. pp. 82–90.

