# Supplementary figures and images for: Small-scale mobility fostering the interaction networks of Patagonian (Argentina) hunter-gatherers during the Late Holocene: Perspectives from strontium isotopes and exotic items
Source: PLoS One. 2023 Feb 15;18(2):e0281089. doi: 10.1371/journal.pone.0281089 (PMC9931128; doi:10.1371/journal.pone.0281089)

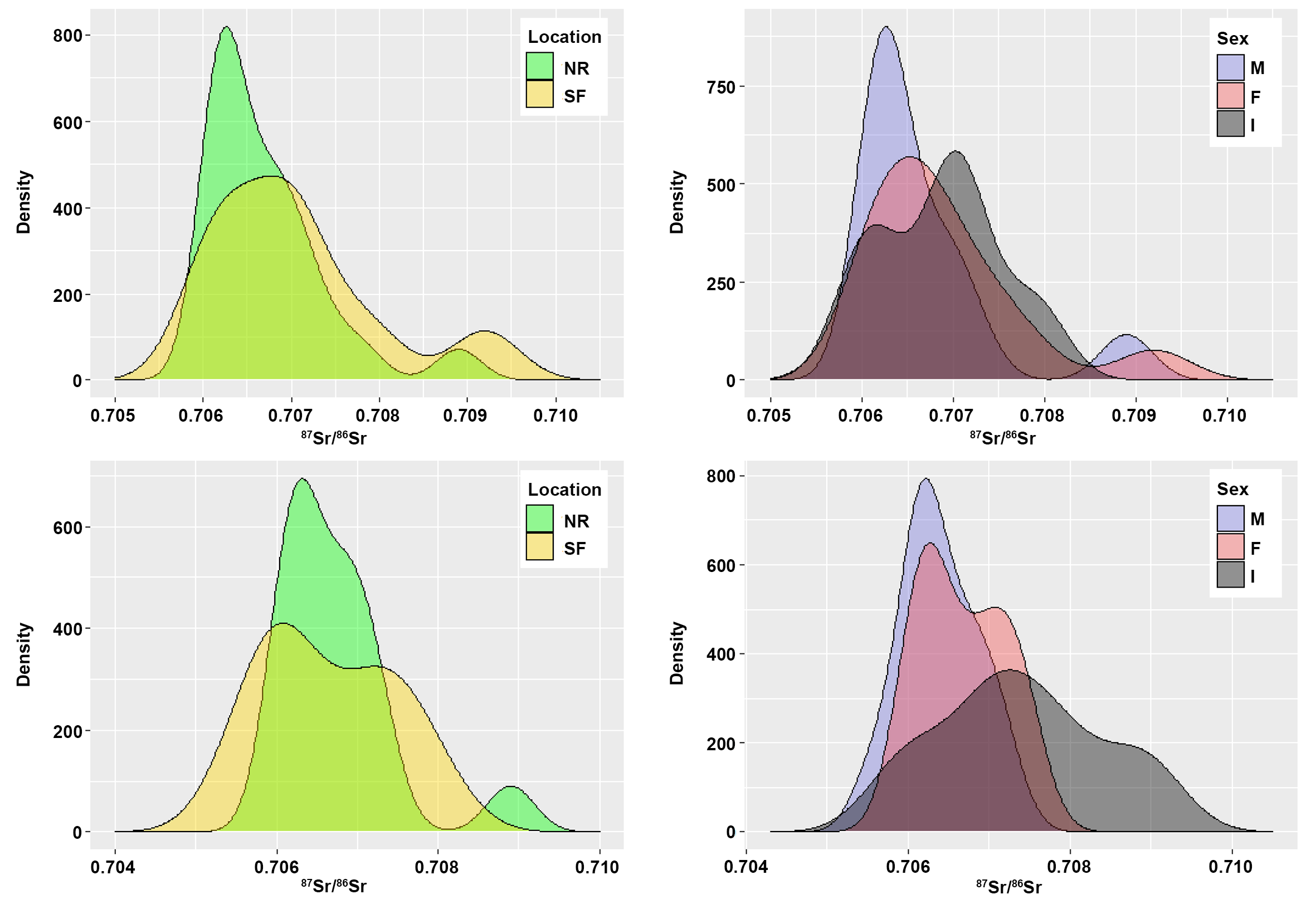

Supplement: S1 Fig — Early (top row) and Late (bottom row) mineralization teeth. (TIF) [file pone.0281089.s001.tif]

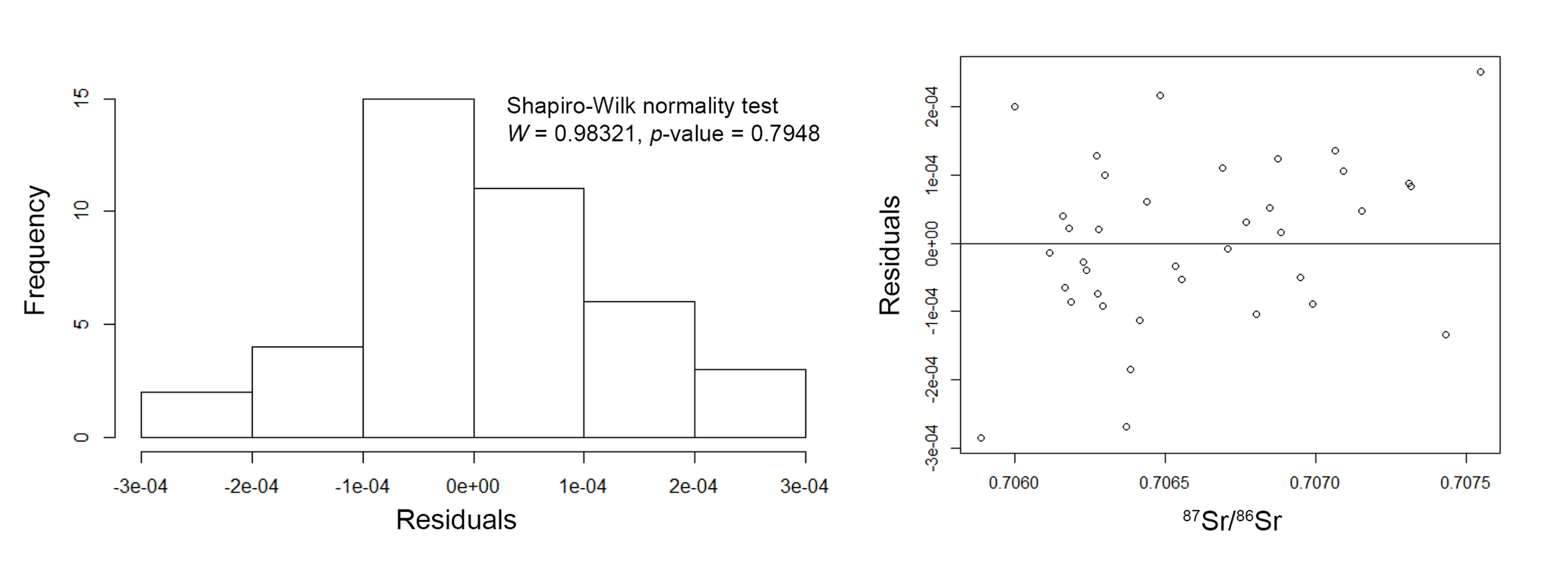

Supplement: S2 Fig — Histogram with normality test (left) and residual plot (right). (TIF) [file pone.0281089.s002.tif]

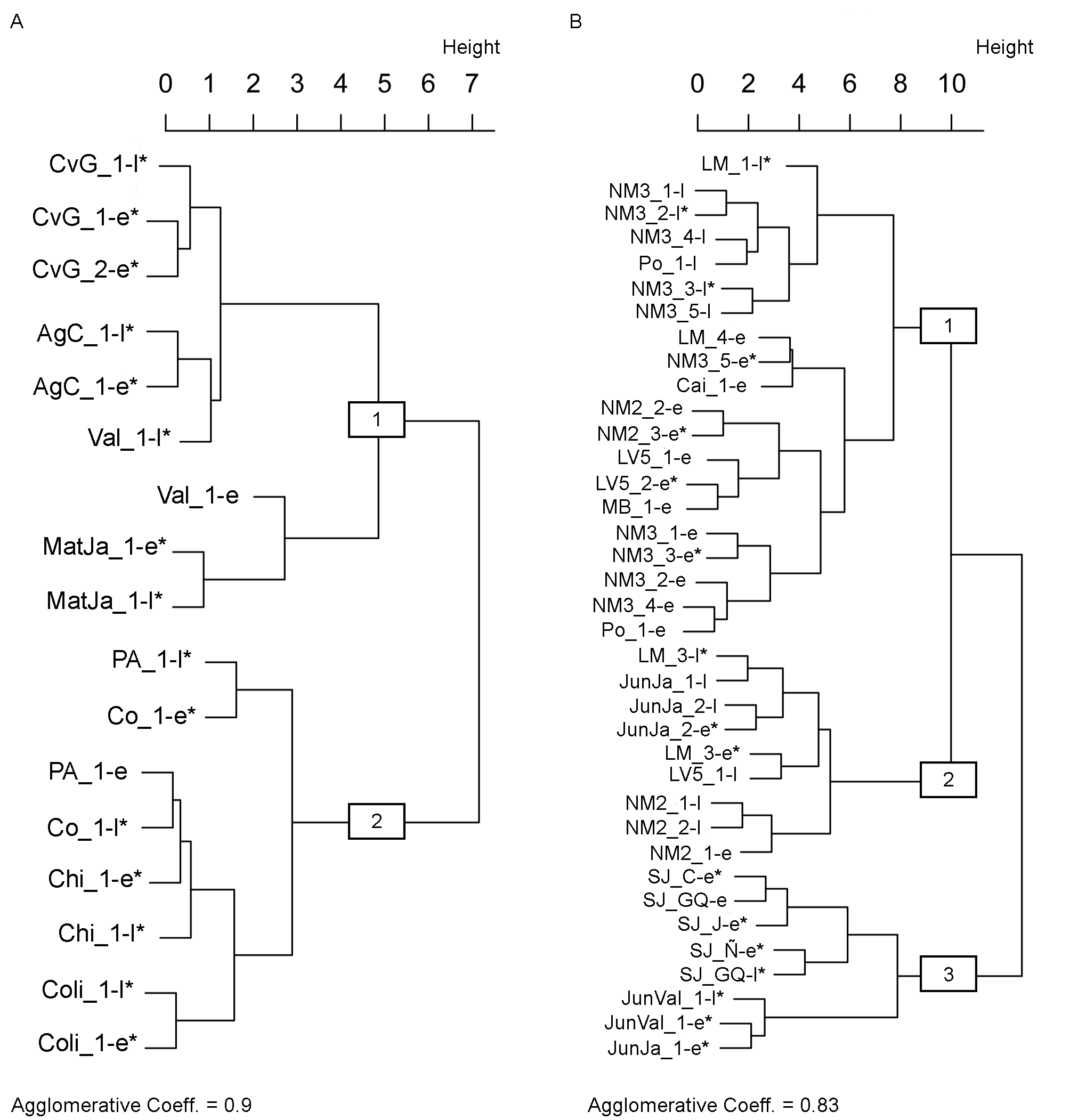

Supplement: S3 Fig — Hierarchical Cluster analyses for SF (A) and NR (B) samples. * signals unidentified drinking water individuals. (TIF) [file pone.0281089.s003.tif]
