# Supplementary material for: Small-scale mobility fostering the interaction networks of Patagonian (Argentina) hunter-gatherers during the Late Holocene: Perspectives from strontium isotopes and exotic items
Source: PLoS One. 2023 Feb 15;18(2):e0281089. doi: 10.1371/journal.pone.0281089 (PMC9931128; doi:10.1371/journal.pone.0281089)
Supplement: S1 Table — (DOCX) [file pone.0281089.s004.docx]

**S1 Table**. **Summary of the ^87^Sr/^86^Sr values from Fig 3**.

| **Distribution** | **n** | **mean** | **± σ** | | **min** | | **Quartile 1** | | **median** | | **Quartile 3** | | **max** | | **outlier** |
| --- | --- | --- | --- | --- | --- | --- | --- | --- | --- | --- | --- | --- | --- | --- | --- |
| *Early teeth by Location* | | | |  | |  | |  | |  | |  | |  |  |
| NR | 22 | 0.7067 | 0.000679 | | 0.7061 | | 0.7062 | | 0.7065 | | 0.7069 | | 0.7078 | | 0.7089 (SJ_Ñ) |
| SF | 9 | 0.7071 | 0.000994 | | 0.7060 | | 0.7063 | | 0.7069 | | 0.7072 | | 0.7079 | | 0.7092 (Val_1) |
| *Early teeth by Sex* | |  |  | |  | |  | |  | |  | |  | |  |
| M | 12 | 0.7069 | 0.000793 | | 0.7062 | | 0.7062 | | 0.7063 | | 0.7069 | | 0.7072 | | 0.7089 (SJ_Ñ) |
| F | 13 | 0.7068 | 0.000782 | | 0.7061 | | 0.7062 | | 0.7067 | | 0.7072 | | 0.7078 | | 0.7092 (Val_1) |
| I | 6 | 0.7068 | 0.000787 | | 0.7060 | | 0.7064 | | 0.7070 | | 0.7071 | | 0.7079 | | - |
| *Late teeth by Location* | | | |  | |  | |  | |  | |  | |  |  |
| NR | 15 | 0.7067 | 0.000722 | | 0.7061 | | 0.7062 | | 0.7065 | | 0.7070 | | 0.7073 | | 0.7089 (SJ_GQ) |
| SF | 8 | 0.7067 | 0.000819 | | 0.7056 | | 0.7061 | | 0.7065 | | 0.7073 | | 0.7079 | | - |
| *Late teeth by Sex* | |  |  | |  | |  | |  | |  | |  | |  |
| M | 10 | 0.7067 | 0.000716 | | 0.7056 | | 0.7061 | | 0.7063 | | 0.7067 | | 0.7072 | | - |
| F | 8 | 0.7067 | 0.000749 | | 0.7061 | | 0.7062 | | 0.7066 | | 0.7071 | | 0.7074 | | - |
| I | 5 | 0.7070 | 0.000922 | | 0.7060 | | 0.7069 | | 0.7073 | | 0.7079 | | 0.7089 | | - |
