# Supplementary material for: Small-scale mobility fostering the interaction networks of Patagonian (Argentina) hunter-gatherers during the Late Holocene: Perspectives from strontium isotopes and exotic items
Source: PLoS One. 2023 Feb 15;18(2):e0281089. doi: 10.1371/journal.pone.0281089 (PMC9931128; doi:10.1371/journal.pone.0281089)
Supplement: S2 Table — Materials from the colonial period are not included (see also Fig 7). (DOCX) [file pone.0281089.s005.docx]

**S2 Table**. **Flow of exotic items within the study area during the Late Holocene**.

| **Zone** | **Site** | **Type** | **Details** | **N** | **Potential**  **Provenance^a^** | **Distance^b^** | **Direct.^c^** | **Exotic^d^** | **Ref.^e^** |
| --- | --- | --- | --- | --- | --- | --- | --- | --- | --- |
| Colorado River lower valley (CRl) | Zoko Andi | Lithic | Chalcedony debitage | 393 | Somuncurá Plateau (12) | 300 | NE | BAMB | 1, 2 |
|  |  | Lithic | Chrysocolla beads | 1 | Lihuel Calel Hills (3)/Chanquín (4) | 265/320 | SE/NE | BAMB | 3, 2 |
|  |  | Malacological | Beads (*Diplodon chilensis*) | 4 | Negro River (5) | 118 | NE | WAMB | 3, 2 |
|  | El Puma 3 | Lithic | Brown silex artifacts | 2 | Yahmoc-Talagapa (11) | 480 | NE | BAMB | 4, 2 |
|  |  | Lithic | Chalcedony debitage | 4 | Somuncurá Plateau (12) | 320 | NE | BAMB | 4, 2 |
|  | Loma Ruiz 1 | Lithic | Chalcedony debitage | 28 | Somuncurá Plateau (12) | 350 | NE | BAMB | 5, 2 |
|  | El Tigre | Lithic | Chalcedony debitage | 652 | Somuncurá Plateau (12) | 350 | NE | BAMB | 6, 7, 2 |
|  | Campo Monaldi | Lithic | Chalcedony debitage | 1 | Somuncurá Plateau (12) | 340 | NE | BAMB | 1, 2 |
| Negro River middle valley (NRm) | La Toma | Lithic | Basalt artifact | 1 | Somuncurá Plateau (12) | 160 | N | BAMB | 8 |
|  |  | Mineral | Chrysocolla beads | 11 | Lihuel Calel Hills (3)/Chanquín (4) | 150/165 | S/N | BAMB | 8 |
|  |  | Metal | Copper pendant | 1 | Lihuel Calel Hills (3) | 150 | S | BAMB | 8, 9 |
|  |  | Malacological | Shells (Volutidae indet.) | 3 | Atlantic coast (1) | 170 | NNW | BAMB | 10 |
|  |  | Malacological | Beads (Mollusca indet.) | 124 | Atlantic coast (1) | 170 | NNW | BAMB | 8 |
|  | Angostura 1 | Vegetable | Endocarp (*Sarcomphalus mistol*) | 1 | Central Argentina Hills (Córdoba/San Luis) (2) | 700 | S | BeAMB | 11 |
|  |  | Malacological | Beads (Mollusca indet.) | 8 | Atlantic coast (1) | 85 | N | WAMB | 12, 10 |
|  |  | Lithic | Chalcedony debitage | 6 | Somuncurá Plateau (12) | 200 | NE | BAMB | 12 |
|  |  | Lithic | Obsidian debitage | 1 | Portada Covunco (7)/Cantera Lolog (8)/Sacanana (10)/Yahmoc-Talagapa (11) | 320 | NE | BAMB | 12 |
|  | Pomona | Lithic | Chalcedony debitage | 4 | Somuncurá Plateau (12) | 190 | NNE | BAMB | 8 |
|  |  | Lithic | Basalt artifact | 1 | Somuncurá Plateau (12) | 150 | NNE | BAMB | 8 |
|  |  | Malacological | Beads (Mollusca indet.) | 32 | Atlantic coast (1) | 150 | NNW | BAMB | 8 |
|  | Negro Muerto 3 | Malacological | Shells (*Mytilus* sp.) | 2 | Atlantic coast (1) | 105 | NNW | WAMB | 13, 10 |
|  |  | Vegetable | Starch and phytoliths (*Zea mays*) | present | South of Mendoza (6) | 600 | SE | BeAMB | 13 |
|  | Negro Muerto | Lithic | Chalcedony debitage | 2 | Somuncurá Plateau (12) | 170 | NE | BAMB | 12 |
|  |  | Malacological | Shells (Volutidae indet.) | 1 | Atlantic coast (1) | 110 | NNW | WAMB | 12 |
|  | Colforta 1 | Lithic | Chalcedony debitage | 2 | Somuncurá Plateau (12) | 190 | NNE | BAMB | 8 |
|  | Ojo de Agua | Lithic | Chalcedony debitage | 1 | Somuncurá Plateau (12) | 170 | NE | BAMB | 12 |
|  | Rincón del Palo | Lithic | Chalcedony debitage | 2 | Somuncurá Plateau (12) | 150 | NE | BAMB | 12 |
|  | El Sifón | Lithic | Obsidian instrument | 1 | Portada Covunco (7)/Cantera Lolog (8)/Sacanana (10)/Yahmoc-Talagapa (11) | 315 | NE | BAMB | 8, 14 |
|  | Arata | Lithic | Andesite instrument | 1 | Somuncurá Foothills (12) | 160 | NNE | BAMB | 8 |
|  | Conesa | Malacological | Shells (Volutidae indet.) | 1 | Atlantic coast (1) | 85 | N | WAMB | 12 |
|  | Loma de los Muertos | Malacological | Shells (Volutidae indet.) | 19 | Atlantic coast (1) | 80 | N | WAMB | 15, 10 |
|  | Oeste de Negro Muerto^1^ | Malacological | Shells (Volutidae indet.) | 7 | Atlantic coast (1) | 110 | N | WAMB | 12 |
| Negro River lower valley (NRl) | Col. Vignati (Laguna del Juncal) | Malacological | Shells (*Adelomelon* sp.) | 3 | Atlantic coast (1) | 30-60 | NW | Regional | 10 |
|  | S/N (Laguna del Juncal) | Malacological | Shells (Volutidae indet.) | 4 | Atlantic coast (1) | 25 | NW | Regional | 16, |
| Closed Basins and Plains (BP) | Trapalcó 1 y 2 | Malacological | Shells (*D*. *chilensis*) | 2 | Negro River (5) | 70 | S | WAMB | 17 |
| Eastern Somuncurá Foothills (SFe) | Cueva Galpón | Vegetable | Cane (*Chusquea sp.)* | 1 | Peri-Andes (9) | 420 | E | BAMB | 18 |
|  |  | Malacological | Beads (Mollusca indet.) | 10 | Atlantic coast (1) | 50 | W | Regional | 18 |
|  |  | Metal | Copper pendant | 1 | Lihuel Calel Hills (3) | 350 | S | BAMB | 9 |
|  | Tembrao | Malacological | Shells (Volutidae) | 2 | Atlantic coast (1) | 100 | W | WAMB | 17; 10 |
|  |  | Malacological | Shells (*D*. *chilensis*) | 2 | Negro River (5) | 175 | SW | BAMB | 19 |
|  | Recintos de Aguada | Malacological | Shells (*D*. *chilensis*) | 1 | Negro River (5) | 120 | NNE | WAMB | 17 |
| Limay River middle valley  (LRm) | Rincón Chico 2/87 | Malacological | Shells (Mollusca indet.) | 4 | Pacific/Atlantic coast (1) | 290/420 | E/WNW | BAMB | 20, 21 |
|  | Piedra del Águila 11 | Malacological | Shells (Mollusca indet.) | 2 | Pacific/Atlantic coast (1) | 285/425 | E/WNW | BAMB | 22 |
|  | Epullán Chica | Malacological | Shells (Mollusca indet.) | 1 | Pacific/Atlantic coast (1) | 280/430 | E/WNW | BAMB | 23 |
| Limay River upper valley-Nahuel Huapi  (LRl-NH) | Casa de Piedra Ortega | Malacological | Shells (*Buccinanops globulosus*) | 1 | Atlantic coast (1) | 470 | E | BAMB | 24, 21 |
|  |  | Malacological | Shells (*Mytilus* sp.) | 3 | Pacific/Atlantic coast (1) | 260/470 | W/E | BAMB | 24, 21 |
|  | Los Cipreses | Malacological | Shells (Mollusca indet.) | 12 | Pacific/Atlantic coast (1) | 205/520 | W/E | BAMB | 21, 25 |
|  |  | Malacological | Shells (*Adelomelon brasiliana*) | 3 | Atlantic coast (1) | 520 | E | BAMB | 21, 25 |
|  | Puerto Tranquilo 1 | Malacological | Beads and shells (Mollusca indet.) | 43 | Pacific/Atlantic coast (1) | 190/540 | W/E | BAMB | 25, 21 |
|  | Cueva Traful III | Malacological | Beads and shells (Mollusca indet.) | 2 | Pacific/Atlantic coast (1) | 230/505 | W/E | BAMB | 21 |
|  | Cuyín Manzano | Malacological | Shells (*Mytilus*sp.) | 1 | Pacific/Atlantic coast (1) | 220/505 | W/E | BAMB | 21 |
|  | Arroyo Corral 1 | Malacological | Shells (Veneridae indet.) | present | Pacific/Atlantic coast (1) | 230/495 | W/E | BAMB | 25 |
|  | Valle Encantado 1 | Malacological | Shells (Mollusca indet.) | 20 | Pacific/Atlantic coast (1) | 225/500 | W/E | BAMB | 26, 21 |
|  | El Manantial 1/88 | Malacological | Beads and shells (Mollusca indet.) | present | Pacific/Atlantic coast (1) | 255/465 | W/E | BAMB | 27 |
|  | Alero Nestares | Malacological | Shells (Mollusca indet.) | 2 | Pacific/Atlantic coast (1) | 260/470 | W/E | BAMB | 28 |
| Southwest Río Negro (Sw-RN) | Cueva Comallo 1 | Malacological | Beads and shells (Mollusca indet.) | present | Pacific/Atlantic coast (1) | 285/445 | W/E | BAMB | 29 |
| San Matías Gulf (SMG) | West and NW^2^ | Lithic | Obsidian artifacts | 12 | Yahmoc-Talagapa (11) | 230-290 | ENE | BAMB | 30 |
|  | West and NW^3^ | Lithic | Obsidian artifacts | 4 | Sacanana (10) | 290-370 | ENE | BAMB | 30 |
|  | West and NW^4^ | Lithic | Obsidian artifacts | 3 | Cantera Lolog (8) | 550 | ESE | BAMB | 30 |
|  | West and NW^5^ | Lithic | Obsidian artifacts | 2 | Telsen (13) | 160-200 | NE | BAMB | 30 |
|  | Punta Pórfido | Lithic | Obsidian artifacts | 1 | Portada Covunco (7) | 550 | SE | BAMB | 31, 32 |

Materials from the post-contact period are not included (see also Fig 7). ^a^For cases with multiple potential sources only the closest ones are included. ^b^Approximate straight distance (km) between the most probable potential source/s and the finding site calculated with Google Earth. Numbers between parentheses refer to Fig 7. ^c^Cardinal direction from the most probable potential source/s to the location of the finding site. ^d^Categories are based on source-finding site distance (see Whallon 2006). Local: <10 km; Regional: 10-50 km; Exotic within adjacent maximal band (WAMB): 50-123km; Exotic between adjacent maximal band (BAMB): 123-600 km; Exotic beyond adjacent maximal band (BeAMB): >600 km. ^1^Salitral de la Victoria, San José 2, San José 3, La Victoria 3, San León. ^2^Bahía Final 110, Saco Viejo, San Antonio Oeste, El Fuerte, Bahía Pozos, Playas Doradas. ^3^Saco Viejo, Piedras Coloradas, Islote Lobos, Bahía Lobos. ^4^Saco Viejo, El Buque Sur. ^5^El Buque Sur, Bahía Pozos.

^e^Table references

1. Santos Valero F. La gestión de rocas a través del tiempo: aportes al estudio de la tecnología lítica durante el Holoceno medio y tardío en el valle inferior del río Colorado (provincia de Buenos Aires). PhD Thesis, FACSO-UNICEN. 2017. Available from https://www.ridaa.unicen.edu.ar/xmlui/handle/123456789/1688.

2. Martínez GA. Síntesis de los sitios y localidades arqueológicas. Resumen del modelo propuesto para el área de estudio. In: Martínez GA, editor. Arqueología de cazadores-recolectores del curso inferior del Río Colorado (provincia de Buenos Aires, Argentina): Aportes al conocimiento de las ocupaciones humanas pampeano-patagónicas. Olavarría: INCUAPA-CONICET; 2017. pp. 45-67.

3. Flensborg GA. Análisis tecnomorfológico de adornos, cuentas y otros ornamentos. In: Martínez GA, editor. Arqueología de cazadores-recolectores del curso inferior del Río Colorado (provincia de Buenos Aires, Argentina): Aportes al conocimiento de las ocupaciones humanas pampeano-patagónicas. Olavarría: INCUAPA-CONICET; 2017. pp. 230-254.

4. Santos Valero F. Contribuciones al conocimiento de la tecnología lítica en el valle inferior del río Colorado: sitio El Puma 3. Intersecciones en Antropología 2015; 16: 237-251.

5. Armentano G, Martínez GA, Landini C. El sitio Loma Ruiz 1: Estrategias tecnológicas líticas durante el Holoceno Tardío Inicial en la transición Pampeano-Patagónica oriental. Revista del Museo de Antropología 2013; 6: 21-40.

6. Armentano G. Arqueología del Curso Inferior del río Colorado. Estudio tecnológico de las colecciones líticas de Norpatagonia oriental durante el Holoceno tardío. Departamentos de Villarino y Patagones, Provincia de Buenos Aires, Argentina. PhD Thesis, FACSO-UNICEN. 2012.

7. Santos Valero F, Armentano G. La tecnología lítica. In: Martínez GA, editor. Arqueología de cazadores-recolectores del curso inferior del Río Colorado (provincia de Buenos Aires, Argentina): Aportes al conocimiento de las ocupaciones humanas pampeano-patagónicas. Olavarría: INCUAPA-CONICET; 2017. pp. 121-145.

8. Mange E. Investigaciones arqueológicas en la margen sur del valle medio-superior del río Negro (provincia de Río Negro). PhD Thesis, FCNyM-UNLP. 2019. Available from: http://sedici.unlp.edu.ar/handle/10915/80578

9. Morita, MM, Zilio L, Bilmes GM. Composition and possible provenance of metallic archaeological objects found in Patagonia argentina. Journal of Arch. Science: Reports 2020; 33 p. 102575. [doi: 10.1016/j.jasrep.2020.102575](https://doi.org/10.1016/j.jasrep.2020.102575).

10. Di Lorenzo M, Mange E, Hammond H, Prates L. El uso de moluscos marinos entre grupos cazadores recolectores del interior norpatagónico en el Holoceno tardío (provincia de Río Negro, Argentina). Arqueología 2022; 28(1): 9926.

11. Capparelli A, Prates, L. Identificación específica de frutos de algarrobo (*Prosopis* spp., Fabaceae) y mistol (*Ziziphus* *mistol*, Rhamnaceae) en un sitio arqueológico de Patagonia. In: Pochettino, ML, Ladio, AH; Arenas, PM, editors. Traditions and transformations in Ethnobotany. San Salvador de Jujuy: CYTED; 2010. pp. 13-19.

12. Prates, L. Los antiguos habitantes del río Negro desde una visión arqueológica. Buenos Aires: Sociedad Argentina de Antropología; 2008.

13. Prates L, Serna A, Mange E, López L, Romano V, Di Lorenzo M, Saghessi D, González L. Ocupaciones residenciales y entierros humanos en Negro Muerto 3 (valle del río Negro, Norpatagonia). Magallania (Punta Arenas), 2019; 47(1): 159-176.

14. Miotti L, Terranova E, Barberena R, Hermo D, Giesso M, Glascock MD. Geochemical sourcing of obsidian fishtail points: studies for the Somuncurá Plateau (Río Negro, Argentina). In: Miotti L, editor. Southbound: Late Pleistocene Peopling of Latin America. Texas: Center for Study of the First Americans; 2012. pp. 127-132.

15. Prates L, Di Prado V, Mange E, Serna A. Sitio Loma de los Muertos: Múltiples ocupaciones sobre un médano del este de Norpatagonia (Argentina). Magallania (Punta Arenas), 2010; 38(1): 165-181.

16. Bórmida M. Cementerios indígenas prehispánicos en la zona de la Laguna del Juncal. Anales del Museo Nahuel Huapi Perito Dr. Francisco P. Moreno, 1950; 2:101–108.

17. Prates L, Mange E. Paisajes de tránsito y estaciones en las planicies y bajos del centro-este de Norpatagonia. Relaciones de la Sociedad Argentina de Antropología, 2016; 41: 217-236.

18. Carden N, Prates L. Pinturas rupestres en un espacio funerario: El caso del sitio Cueva Galpón (Noreste de Patagonia). Magallania (Punta Arenas), 2015; 43(1): 117-136.

19. Mange E, Di Lorenzo M, González Venanzi L. Los restos faunísticos del sitio arqueológico Tembrao (provincia de Río Negro, Argentina). Revista del Museo de Antropología, 2018; 11(1): 25-34.

20. Crivelli Montero, E. y M. Ramos. Hallazgos especiales de Rincón Chico 2/87. In: Crivelli Montero E, Fernández M, Ramos M. Arqueología de rescate en Rincón Chico. Buenos Aires: Dunken; 2009. pp. 205-213.

21. Silveira M, López L, Pastorino G. Movilidad, redes de intercambio y circulación de bienes en el sudoeste del Neuquén (Norpatagonia, Argentina). Los moluscos marinos del lago Traful. Intersecciones en antropología, 2010; 11(1): 227-236.

22. Sanguinetti de Bórmida A, Curzio D. Cronología regional, cultural y paleoambiental del área de investigación Piedra del Águila. Praehistoria, 1996; 2: 280-290.

23. Crivelli Montero E, Guillermo A, Fernández FJ. Hallazgos especiales del sitio arqueológico Epullán Chica (holoceno tardío, Neuquén, Argentina). Cuadernos de Antropología, 2018; 20: 41-60.

24. Fernández MM, Ramos M. Hallazgos especiales del sitio Casa de Piedra de Ortega, provincia de Río Negro. Anales de Arqueología y Etnología, 2017; 61: 147-164.

25. Hajduk A, Albornoz AM, Lezcano M. Espacio, cultura y tiempo: el corredor bioceánico norpatagónico desde la perspectiva arqueológica. In Navarro Floria P, Delrio W, editors. Cultura y espacio. Araucanía-Norpatagonia. San Carlos de Bariloche: Iidypca; 2011. pp. 262-292.

26. Hajduk A, Albornoz AM. El sitio Valle Encantado I. Su vinculación con otros sitios. Un esbozo de la problemática local diversa del Nahuel Huapi. In Belardi J, Fernández P, Goñi R, Guráieb A, de Nigris M, editors. Soplando en el Viento. Actas de las III Jornadas de Arqueología de la Patagonia. Neuquén: UNCOMA-INAPL, 1999. pp. 371-391.

27. Sanguinetti de Bórmida AC, Curzio D, Crivelli Montero E, Chauvin A. Arqueología de El Manantial, Corralito y Limay Chico (Cuenca del Río Limay. Provincias de Río Negro y del Neuquén). Las campañas de 1995 y 1996. In Belardi J, Fernández P, Goñi R, Guráieb A, de Nigris M, editors. Soplando en el Viento. Actas de las III Jornadas de Arqueología de la Patagonia. Neuquén: UNCOMA-INAPL, 1999. pp. 539-559.

28. Zubimendi MA. Síntesis historiográfica de las investigaciones sobre artefactos arqueomalacológicos de la Patagonia Continental Argentina (1867-2011). In Hammond H, Zubimendi M, editors. Arqueomalacología: abordajes metodológicos y casos de estudio en el Cono Sur. Buenos aires: Vázquez Mazzini, 2015. pp. 217-253.

29. Cordero JA. Explotación animal en el Holoceno del noroeste de la Patagonia argentina Cambios climáticos y transformaciones del comportamiento humano: una primera aproximación. PhD Thesis, FFyL-UBA. 2010. Available from: <http://repositorio.filo.uba.ar/handle/filodigital/1850>.

30. Favier Dubois CM, Stern, C, Cardillo M. Primera caracterización de los tipos de obsidiana presentes en la costa rionegrina. In Salemme M, Santiago F, Álvarez M, Piana, E, Vázquez M, Mansur E., editors. Arqueología de la Patagonia. Una mirada desde el último confín. Ushuaia: Utopías, 2009. pp. 349-359.

31. Alberti J, Cardillo M, Stern C, Favier Dubois, CM. New results concerning expanded networks of obsidian procurement in San Matías Gulf, Río Negro, Patagonia, Argentina. The Journal of Island and Coastal Archaeology, 2016; 11(3): 435-442.

32. Alberti J. Disponibilidad y explotación de materias primas líticas en la Costa de Norpatagonia (Argentina): un enfoque regional. Oxford: Archaeopress Publishing Ltd; 2016.
